# Transcriptional profiling provides insights into metronomic cyclophosphamide-activated, innate immune-dependent regression of brain tumor xenografts

**DOI:** 10.1186/s12885-015-1358-y

**Published:** 2015-05-08

**Authors:** Joshua C Doloff, David J Waxman

**Affiliations:** Department of Biology, Division of Cell and Molecular Biology, Boston University, Boston, USA

**Keywords:** Immunogenic chemotherapy, Microarray, Mouse tumor models, U251 glioblastoma, Innate immunity

## Abstract

**Background:**

Cyclophosphamide treatment on a six-day repeating metronomic schedule induces a dramatic, innate immune cell-dependent regression of implanted gliomas. However, little is known about the underlying mechanisms whereby metronomic cyclophosphamide induces innate immune cell mobilization and recruitment, or about the role of DNA damage and cell stress response pathways in eliciting the immune responses linked to tumor regression.

**Methods:**

Untreated and metronomic cyclophosphamide-treated human U251 glioblastoma xenografts were analyzed on human microarrays at two treatment time points to identify responsive tumor cell-specific factors and their upstream regulators. Mouse microarray analysis across two glioma models (human U251, rat 9L) was used to identify host factors and gene networks that contribute to the observed immune and tumor regression responses.

**Results:**

Metronomic cyclophosphamide increased expression of tumor cell-derived DNA damage, cell stress, and cell death genes, which may facilitate innate immune activation. Increased expression of many host (mouse) immune networks was also seen in both tumor models, including complement components, toll-like receptors, interferons, and cytolysis pathways. Key upstream regulators activated by metronomic cyclophosphamide include members of the interferon, toll-like receptor, inflammatory response, and PPAR signaling pathways, whose activation may contribute to anti-tumor immunity. Many upstream regulators inhibited by metronomic cyclophosphamide, including hypoxia-inducible factors and MAP kinases, have glioma-promoting activity; their inhibition may contribute to the therapeutic effectiveness of the six-day repeating metronomic cyclophosphamide schedule.

**Conclusions:**

Large numbers of responsive cytokines, chemokines and immune regulatory genes linked to innate immune cell recruitment and tumor regression were identified, as were several immunosuppressive factors that may contribute to the observed escape of some tumors from metronomic CPA-induced, immune-based regression. These factors may include useful biomarkers that facilitate discovery of clinically effective immunogenic metronomic drugs and treatment schedules, and the selection of patients most likely to be responsive to immunogenic drug scheduling.

**Electronic supplementary material:**

The online version of this article (doi:10.1186/s12885-015-1358-y) contains supplementary material, which is available to authorized users.

## Background

Metronomic chemotherapy utilizes drug dosages that are lower, and are given at regular, more frequent intervals than conventional maximum tolerated dose regimens, without extended rest periods [1-4]. Clinical trials of metronomic therapy commonly use cyclophosphamide (CPA; 43% of all such trials) [5], which is typically given on a low dose daily schedule [6,7]. Low dose daily metronomic dosing has shown promise in terms of improved therapeutic activity and reduced host toxicity compared to maximum tolerated dose chemotherapy, however, large randomized trials are required to definitively establish its therapeutic advantages. Metronomic dosing is widely thought to act by an anti-angiogenic mechanism [1,8], reflecting the preferential sensitivity of tumor endothelial cells to low doses of CPA and several other cytotoxics [9]. However, there is increasing evidence for important effects of metronomic chemotherapy on other tumor-associated cells, in particular immune cells [3,10].

Innate immunity, rather than anti-angiogenesis, can be a key mechanism leading to major regression by metronomic chemotherapy of some large, established tumors [11], as seen in work from this laboratory in implanted brain tumor models when CPA is delivered using an intermittent (every 6-day) metronomic schedule [12-15]. Macrophages, natural killer cells and dendritic cells and other bone marrow-derived innate immune cells were increased in both 9L and U251 gliomas implanted in adaptive immune (T cell and B cell)-deficient *scid* mice. Similar responses were achieved in immunocompetent mice, where syngeneic GL261 gliomas can be completely regressed by metronomic CPA delivered on a 6-day schedule [12,16]. Several cytokines and chemokines associated with mobilizing innate immune response cells [17,18] were also identified in these models of metronomic CPA-induced regression, including CXCL14, IL-12β, and CXCL12/SDF1α. In contrast, when the 6-day repeating metronomic CPA treatment was tested in NOD-scid-gamma mice, which unlike *scid* mice, have deficiencies in the innate immune system [19,20], tumor growth delay with eventual stasis, but not tumor regression, was achieved [12].

Intermittent metronomic CPA treatment preferentially eliminates immunosuppressive CD11b^+^Gr1^+^ myeloid-derived suppressor cells (MDSCs) from bone marrow and spleen of glioma-bearing mice [14]. Tumor regression in our glioma models is not, however, a secondary response to the relief of innate MDSC suppression of innate NK cells [21] or to the adaptive Treg cell-based suppression of innate and adaptive cytotoxic lymphocytes reported for other metronomic regimens [22-24]. Rather, it is a direct consequence of the mobilization of innate immune cells and their recruitment to and infiltration of the chemotherapy-damaged tumors. Further supporting the essential role of the innate immune system, NK cell depletion by anti-asialo-GM1 antibody treatment increases tumor take rates and stimulates tumor growth in various human and mouse tumor models, including allogeneic YAC-1 tumors, which do not grow without NK depletion [25], and renders the regression of implanted GL261 gliomas incomplete following metronomic CPA treatment [12,16]. Withdrawal of anti-asialo-GM1 antibody treatment while continuing the every 6-day metronomic CPA regimen led to repopulation of the tumors by NK cells and resumption of tumor regression [12].

The mechanisms by which metronomic CPA activates and mobilizes anti-tumor innate immune cells and then recruits them to the drug-treated tumors are unknown. These mechanisms could involve tumor cell death and DNA damage or cell stress response pathways that activate a targeted immune response resulting in tumor clearance. Further, predictive factors of response have been elusive, making it difficult to optimize the dose and frequency of metronomic drug treatment [4,5,7,26] or to predict which tumors (and which patients) are likely to be responsive to immunogenic metronomic scheduling, and which ones are not [27]. To address these issues, we carried out genome-wide transcriptional profiling of untreated and metronomic CPA-treated human U251 tumor xenografts using human microarrays. This enabled us to identify tumor cell-specific factors that may elicit anti-tumor innate immunity. It also allowed us to characterize in a comprehensive and unbiased manner the anti-tumor innate immune response, including immune-based signaling pathways important for activating and mobilizing a targeted immune response. We also conducted transcriptional profiling of metronomic CPA-treated rat 9L and human U251 tumor xenografts using mouse microarrays. We could thus validate metronomic CPA-responsive mouse genes whose expression was previously found to be altered in the tumor compartment [12-16], as well as identify many previously unidentified host immune factors, cell types, and signaling molecules important for immune recruitment and tumor regression. Together, these findings elucidate metronomic CPA-responsive gene networks and their upstream regulators, and provide important insights into how intermittent metronomic CPA scheduling activates potent anti-tumor innate immunity leading to prolonged tumor regression.

## Methods

### Cell lines and reagents

CPA monohydrate was purchased from Sigma Chemical Co. (St. Louis, MO). Fetal bovine serum (FBS) and cell culture media were purchased from Invitrogen-Life Technologies (Carlsbad, CA). Glioma cell lines were authenticated by and obtained from the following sources: human U251 glioblastoma from the Developmental Therapeutics Program Tumor Repository (National Cancer Institute, Frederick, MD), and rat 9L gliosarcoma from the Neurosurgery Tissue Bank (UCSF, San Francisco, CA). Cells were grown at 37°C in a humidified, 5% CO_2_ atmosphere; U251 cells were grown in RPMI 1640 and 9L in DMEM culture medium, both of which contained 10% FBS, 100 units/ml penicillin and 100 μg/ml streptomycin.

### Tumor xenografts

Male ICR/Fox Chase immune deficient *scid* mice 5–6 weeks old (24–26 g) (Taconic Farms, Germantown, NY) were housed in the Boston University Laboratory of Animal Care Facility. Animals were treated using protocols specifically reviewed for ethics and approved by the Boston University Animal Care and Use Committee. 9L cells (4 × 10^6^) or U251 cells (6 × 10^6^) were injected *s.c.* on each posterior flank in 0.2 ml serum-free DMEM using a 0.5-inch 29-gauge needle and a 1 ml insulin syringe. Tumor areas (length × width) were measured twice weekly using Vernier calipers (VWR, Cat. #62379-531) and tumor volumes were calculated based on Vol = (π/6)*(L*W)^3/2^. Tumors were monitored and treatment groups were normalized (each tumor volume set to 100%) once average tumor volumes reached ~500 mm^3^. Mice were treated with CPA monohydrate on an intermittent metronomic schedule by i.p. injection at 140 mg CPA/kg body weight (BW) every 6 days [11], with the dose reported here based on the non-hydrate molecular weight of 261. Tumor sizes and mouse body weights were measured at least twice weekly. Tumor growth rates prior to drug treatment were similar among all normalized groups. CPA-treated tumors were collected 6 days after either the 2^nd^ or the 3^rd^ CPA treatment cycle (U251 tumors) or 6 days after the 4^th^ treatment cycle (9L tumors), i.e., treatment days 12, 18 and 24, respectively. Drug-free control tumors were collected on days 6, 12, and 18 (U251) and on days 0 and 10 (9L), where day 0 is the first day of drug treatment.

### RNA processing and microarray analysis

Total RNA was extracted from tumor tissue using TRIzol (Invitrogen). Only high quality RNA was used in this study, as determined by Agilent Bioanalyzer (RIN value 8 or higher using Agilent Nano-Lab Chip Kit; Agilent Technologies, Santa Clara, CA). Randomized RNA pools (two independent pools per treatment group; biological replicates) were generated for both untreated and metronomic CPA-treated tumor samples by randomly distributing tumor RNA samples into pools, with each pool comprised of the following: 7–8 untreated 9L tumor RNAs (pools JD1, JD2), 4 CPA-treated 9L tumor RNAs collected 6 days after the 4^th^ CPA treatment (pools JD3, JD4), 8 untreated U251 tumor RNAs (pools JD5, JD6), 6–7 CPA-treated U251 tumor RNAs collected 6 days after the 2^nd^ CPA injection (pools JD9, JD10), or collected 6 days after the 3^rd^ CPA injection (pools JD7, JD8). Each pool was prepared by combining equal amounts of RNA from each of the individual tumors comprising the pool, to give a total of 7.5 μg tumor RNA. RNA concentrations were determined for each pool by Nanodrop analysis (Thermo Fisher Scientific Inc., Waltham, MA) and the RNA quality (RIN number) was reconfirmed by Bioanalyzer analysis. Tumor RNA pools were used in a total of 10 two-color, metronomic CPA-treated vs. untreated control tumor hybridization microarrays by pairing the following pools: JD1 with JD3, and JD2 with JD4 (9L tumors; comparison A); JD5 with JD9, and JD6 with JD10 (U251 tumors; comparison B); JD5 with JD7, and JD6 with JD8 (U251 tumors; comparison C). Alexa 555-labeled and Alexa 647-labeled amplified RNA samples were hybridized to Agilent Whole Mouse Genome oligonucleotide microarrays (4 × 44 K platform (version 2) (Agilent Technology; platform GPL10333, array design #026655, containing 39,429 unique probes) for 9L tumors (comparison A, above) and U251 tumors (comparisons B and C, above) to probe for changes in expression of host cell (mouse) RNAs. The same U251 tumor RNA pools (comparisons B and C, above) were also analyzed on Agilent Whole Human Genome oligonucleotide microarrays (4 × 44 K platform, version 2; platform GPL10332, array design #026652, containing 34,127 unique probes) to probe for changes in expression of (human) tumor RNAs. Biological replicates were analyzed with dye swaps to eliminate dye bias, as described elsewhere [28,29], giving a total of 6 mouse arrays and 4 human arrays.

### Microarray data and statistical analysis

Analysis of TIFF images of each scanned slide using Agilent’s feature extraction software, calculation of linear and LOWESS normalized expression ratios and initial data analysis and *p-*value calculation using Rosetta Resolver (version 5.1, Rosetta Biosoftware, Seattle, WA) [30]) were carried out by Dr. Alan Dombkowsky at the Wayne State University microarray facility (Detroit, MI) as described [28,31]. The Rosetta error model provides a gene-specific estimate of error by incorporating two elements: a technology-specific estimate of error and an error estimate derived from replicate arrays [30]. The technology-specific component utilizes an intensity-dependent model of error derived from numerous self-self hybridizations. By including the technology-specific estimate, the Rosetta error model avoids false positives that occur from under-estimation of error when a small number of replicate arrays are available, thus increasing the statistical power equivalent to that which would be obtained with at least one additional replicate. Furthermore, a log-ratio error estimate was derived in the Rosetta error model from the individual error estimates of each sample (color) used in the co-hybridization. Then, for each feature an average log ratio and associated *p*-value was obtained from replicate measurements (arrays) using the Rosetta error model error-weighted averaging method, which weighs the ratio of each sample inversely proportional to the variance of that sample. This gives an averaged ratio with the smallest possible error. The Rosetta error model has superior accuracy in detecting and quantifying relative gene expression when compared to other statistical methods commonly used in microarray analysis, as shown by validation with spike-in experiments [32]. The full set of normalized expression ratios and p-values is available at the Gene Expression Omnibus web site (http://www.ncbi.nlm.nih.gov/geo) as GEO series GSE60864, GSE60866, and GSE60867 (GEO SuperSeries GSE60913). For analyses, both human- and mouse array-derived gene lists were generated based on |fold change| > 1.5 and p-value < 10^−4^; these cutoff values balanced the need to minimize false positives while maximizing microarray signal:noise. To determine microarray probe species specificity, the complete sets of human and mouse microarray probes (60 nt each) were analyzed by BLAT [33] in comparison to human and mouse genome sequences (hg19 and mm9) and RefmRNA and mRNA sequences downloaded from the UCSC genome browser. A high degree of species specificity was apparent: 91.3% of the human microarray probes matched human RefmRNA or mRNA sequences (‘match’ defined as sequence identity (match score) of ≥ 50 nt of the 60 nt microarray probe), while only 10.2% matched mouse RNA sequences. Similarly, 90.3% of the mouse array probes matched mouse RefmRNA and mRNA sequences (match score ≥ 50 nt), while only 9.9% matched human RNA sequences.

### Gene ontology and upstream regulator analysis

The DAVID annotation tool [34] was used to analyze sets of metronomic CPA-responsive genes identified at each time point and in each tumor model to discover functional gene clusters, based on gene ontology and other gene annotations, that show significant enrichment (enrichment score ≥1.3, equivalent to p ≤ 0.05). The upstream pathway analysis module of Ingenuity Pathway Analysis (IPA) (Build 320386 M, Version 21249400) was used to calculate upstream regulator enrichments and to determine whether the regulators identified are either in an activated or an inhibited state [35]. Overlap p-values were calculated by IPA using Fisher’s Exact Test to determine the likelihood that the putative upstream regulator is in fact an upstream regulator, based on the significance of the overlap between the known targets of each putative upstream regulator and the identified set of regulated genes. Overlap p-values <0.01 are considered significant by IPA; however, we increased the stringency to p < E-04 to focus on those regulators with a high probability for upstream regulation. For each upstream regulator that met these cutoffs, an activation Z-score, calculated by IPA, was determined by comparing the known effect of the regulator on each target gene (activation or suppression) to the observed changes in gene expression. Based on the concordance between these patterns, an activation Z-score was assigned by IPA after correcting for cases where the regulation directions of the dataset and downstream causal edges are skewed, enabling us to infer whether a given upstream regulator was in an activated state (bias-corrected Z-score > 2), an inhibited state (bias-corrected Z-score < −2), or an uncertain state [35]. An overlap p-value < E-04 was also applied when carrying out mechanistic network refinement within IPA. Upstream regulators that were drugs and other exogenous chemicals were excluded from further consideration and are not presented.

## Results

### Impact of metronomic CPA treatment on tumor cell gene expression

Microarray analysis of U251 human tumor xenografts was carried out to identify human tumor cell genes whose expression was either increased or decreased by CPA treatment on a 6-day repeating metronomic schedule. Tumor RNA samples were analyzed on treatment days 12 and 18, i.e., 6 days after the 2^nd^ and 6 days after the 3^rd^ CPA injections, respectively. Day 12 represents an early time point in innate immune cell recruitment and tumor regression, while day 18 is well into the tumor regression response [12]. Tumor transcriptional profiles were assayed using human microarrays containing ~40,000 probes representing ~20,000 human genes. Genes showing significant increases or decreases in expression compared to drug-free controls were identified: expression of 806 genes increased at both time points while 641 genes decreased at both time points. Further, only 8 genes showed opposite regulation at day 12 vs. day 18, indicating a very high consistency of the directionality of responses between time points. Many other genes showed significant changes in expression on day 12 only, or on day 18 only. A completed listing of all regulated microarray probes, and their associated gene names and annotations, expression ratios, p-values and signal intensities is provided in Additional file 1: Table S1. Table 1 presents expression data for select examples of U251 tumor cell genes whose responses to metronomic CPA are beneficial to the overall therapeutic response, as well as genes whose responses are undesirable, e.g., induction of the tumor-promoting MMP13, the immune-inhibitory adhesion molecule CEACAM1, and the pro-metastatic factors LAMP3/CD208 and ACP5.

**Table 1:** **Examples of U251 human tumor cell genes that constitute beneficial responses (A) or undesirable responses (B) to metronomic CPA treatment**

A. Beneficial responses						
Gene	U251 (day 12)		U251 (day 18)		Pro-tumor or Anti-tumor Activities	References
	*Fold change*	*p-value*	*Fold change*	*p-value*		
LUM	8.7	*1.3E-06*	14.1	*1.7E-14*	Inhibits tumor cell migration and invasion	[112]
SSTR2	5.5	*7.5E-23*	5.9	*0*	Inhibits glioma proliferation	[113]
IFNB1	5.5	*9.3E-37*	5.9	*2.5E-43*	Pro-apoptotic, anti-proliferative, anti-angiogenic factor; inhibits accumulation of pro-angiogenic tumor-associated neutrophils	[114,115]
ZBP1	4.8	*0*	5.6	*0*	DNA sensor; activates IRFs, NFkB, and innate immunity; interferon-inducible	[116-118]
XAF1	2.8	*2.2E-35*	5.5	*0*	Interferon-inducible, pro-apoptotic	[119]
IFIT3	4.0	*1.7E-37*	5.3	*7.8E-42*	Interferon-inducible, pro-apoptotic	[120]
DMBT1	3.3	*7.0E-15*	5.0	*0*	Tumor suppressor down-regulated in glioblastoma	[121]
DDX58	2.8	*6.5E-43*	4.5	*0*	Induces interferon-I, activates apoptosis	[122]
TNFSF4/OX40L	2.3	*2.5E-08*	3.5	*2.8E-31*	Increases adhesion of activated T cells at tumor site	[123]
CXCL2/MIP2	−4.6	*3.8E-14*	−3.1	*1.3E-09*	Up-regulated in temozolomide-resistant glioma	[124]
CXCR4	−3.6	*7.4E-38*	−5.0	*2.0E-26*	Promotes angiogenesis in glioma	[125,126]
LGR5	−4.7	*3.8E-43*	−5.3	*1.2E-39*	Marker for poor prognosis in glioblastoma	[127]
IL8/CXCL8	−7.6	*1.5E-11*	−5.5	*1.1E-24*	Proinflammatory cytokine; increases tumor angiogenesis, invasion and metastasis; interferon-inducible	[128]
**B.B. Undesirable responses**						
**Gene**	**U251 (day 12)**		**U251 (day 18)**		**Pro-tumor or Anti-tumor Activities**	**References**
	***Fold change***	***p-value***	***Fold change***	***p-value***		
MMP13	10.1	*1.1E-11*	15.4	*0*	Promotes tumor cell proliferation and invasion	[129]
CEACAM1	5.9	*4.7E-29*	13.2	*7.7E-29*	Immune-inhibitory adhesion molecule; interferon-inducible	[94]
LAMP3/CD208	6.4	*1.5E-30*	9.6	*0*	Promotes metastases	[130]
EREG	6.5	*2.3E-25*	8.5	*0*	Binds EGFR and induces glioma cell growth	[95]
IDO1	4.8	*0*	6.5	*0*	Immunosuppressive in human glioblastoma; interferon-inducible	[96]
ACP5	4.5	*2.3E-33*	5.0	*7.2E-39*	Pro-metastatic factor	[131]

Shown are fold-change values (fold increases or decreases in expression compared to drug-free controls) and associated p-values derived from microarray analyses for U251 tumors analyzed on day 12 and day 18 after initial CPA treatment.

DAVID analysis [34] identified functional gene ontology clusters significantly enriched in the sets of U251 tumor cell-expressed genes showing a consistent pattern of increased expression at both CPA time points. Highest enrichments were found for the gene ontology clusters inflammatory/defense response, histone/nucleosome core, cytokine activity and cytokine stimulus, induction/regulation of apoptosis, and positive regulation of the (innate) immune system (Table 2A; Additional file 1: Table S2A). Genes whose expression was decreased at both CPA time points were associated with extracellular signal, cell adhesion, skeletal system and blood vessel development, and extracellular matrix genes (Table 2B; Additional file 1: Table S2B). The top up-regulated gene cluster, inflammatory defense response, included several chemokines and chemokine receptors (*CXCL9, CXCL10, CXCL11*, *CCL5*, *CCL26*, *CCR1)*, interleukins and interleukin receptors (*IL4*, *IL23A, IL1R1, IL17RB, IL20RB*), tumor necrosis factor ligand *TNFSF4*, interferon *IFNB1*, complement components (*C1QB*, *C1S*, *C2*, *C3*, *C4B*, *CFB, CFH*), peroxisome proliferator-activated receptor *PPARG*, and Toll-like receptors *TLR3* and *TLR4*.

**Table 2:** **Enriched clusters of gene annotation terms for U251 (human) tumor genes up-regulated (A) or down-regulated (B) by metronomic CPA treatment**

Cluster name	Cluster enrichment score	Number of genes (top term)	p-value (top term)
**A. Up-regulated tumor gene clusters**			
Inflammatory/defense response to wounding	9.15	63	3.91E-12
Signal peptide/glycoprotein/secreted	6.01	190	7.67E-13
Histone/nucleosome core	5.02	17	3.00E-12
Integral plasma membrane	4.29	83	1.29E-06
Cytokine activity	3.38	20	1.68E-04
Induction/regulation of apoptosis	3.26	31	7.73E-06
Response to bacterium/cytokine stimulus	2.99	21	6.36E-05
Positive regulation of (innate) immune system	2.72	29	1.72E-07
**B. Down-regulated tumor gene clusters**			
Extracellular signal	5.04	81	2.75E-08
Cell adhesion	2.92	37	1.12E-04
Skeletal system development	2.70	22	1.24E-04
Extracellular matrix	2.61	25	1.98E-05
EGF-like	2.58	19	2.58E-06
Blood vessel development	2.54	17	8.45E-04

Analysis was based on genes that respond consistently after both 2 and 3 CPA/6-day treatment cycles (i.e., treatment days 12 and 18) at |fold-change| >1.5 and p-value < 10^4^. Shown are clusters with enrichment scores >2.5 whose top term contains >15 genes. Also shown is the number of genes and p-value for the top term in each cluster. See Additional file 1: Tables S2A and S2B for a more complete listing of significant enrichment clusters and associated gene lists.

### Tumor-specific pathways activated by metronomic CPA treatment

**Figure 1:**
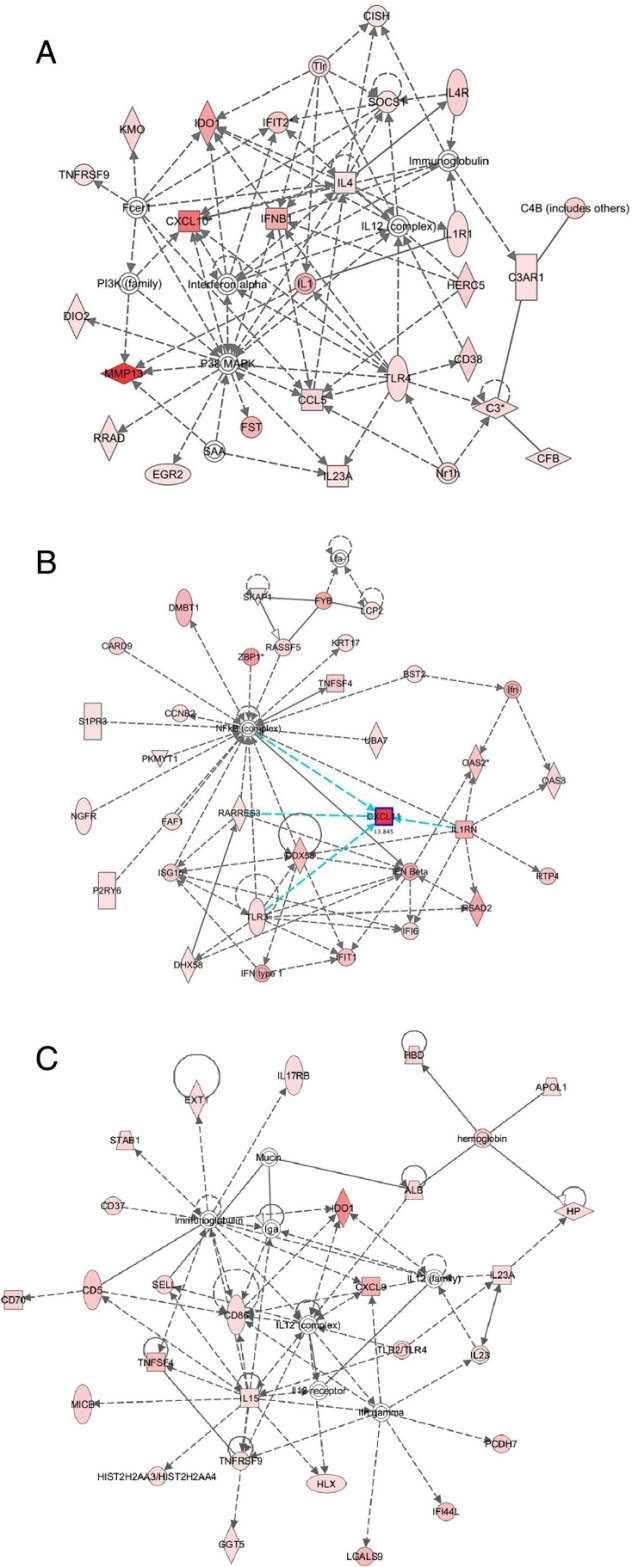
Top networks associated with U251 tumor human genes increased by metronomic CPA treatment on both day 12 and day 18 (late responses), as determined by IPA. **A)** Top network for the human chemokine CXCL10, involved in innate immune activation via toll-like receptor (TLR) and interferon (IFN) response pathways. **B)** Top network for the human chemokine CXCL11, involved in innate immune activation via DNA damage, TLR, IFN, and secretory chemokine/cytokine pathways. **C)** Top network for the human chemokine CXCL9, involved in innate immune activation via toll-like receptor, interleukin, and cell stress ligand MICB response pathways. Deeper shades of red-filled shapes indicate stronger up regulation of the gene by metronomic CPA treatment, as determined by microarray analysis. Solid arrows: protein-DNA interactions; solid lines: protein-protein; dashed arrows: regulation of gene expression; colored: related to highlighted factor(s). Shapes indicate protein family: rectangle: receptor; square: cytokine; triangle: kinase; diamond: enzyme; oval: factor (ie., transcription); concentric circles: complex; circle: other.

Functional gene networks were constructed based on the sets of tumor cell genes whose expression was significantly induced or repressed by metronomic CPA treatment. One such network (Additional file 2: Figure S1), which is activated on day 12 and may contribute to the early anti-tumor actions of CPA, includes many intracellular cell death factors, such as *BIK*, important for mitochondrial rupture, death effector signaling caspases, DNA repair and cell death signaling poly-A ribose polymerases (*PARP10* and *PARP12*), tumor necrosis factor *TNFSF10*, the 20S proteasome, and several cytokeratins, including KRT18, which is released from CPA-treated tumors and is a biomarker for clinical response to therapy [36]. Genes important for extracellular presentation of cellular stresses and activating inflammatory immune responses were also induced at both early (day 12) and late (day 18) CPA treatment times. Pathways involving tumor-expressed extracellular membrane-bound chemokines *CXCL9*, *CXCL10*, and *CXCL11* were identified and show potential interactions in networks translating intracellular damage to extracellular signals that may stimulate immune recruitment and tumor cell death (Figure 1A-C). *CXCL10* increased almost 10-fold over untreated controls at both time points and is centric to a network involving many interferon and innate immune response genes, including *IFNB1*, *TLR4*, and *IDO1*, an immunosuppressive factor (Figure 1A). *CXCL11* expression increased almost 14-fold, and is tied to several chemokines important for extracellular signaling and immune activation in addition to interferon and TLR3 activation (Figure 1B). *CXCL9* was also induced in the metronomic CPA-treated tumor cells in association with other extracellular immune activators: *TNFSF4*, *MICB*, interleukins *IL12*, *IL15, IL23, and IL17RB*, and interferon response genes (Figure 1C). *MICB* is one of two induced MHC class I and DNA damage response-associated activating ligands for the NK cell receptor NKG2D; *MICB* was significantly induced at both CPA time points, while a second such factor, *ULBP2*, was up-regulated at the day 18 time point only (Additional file 1: Table S1).

### Upstream regulator analysis

**Figure 2:**
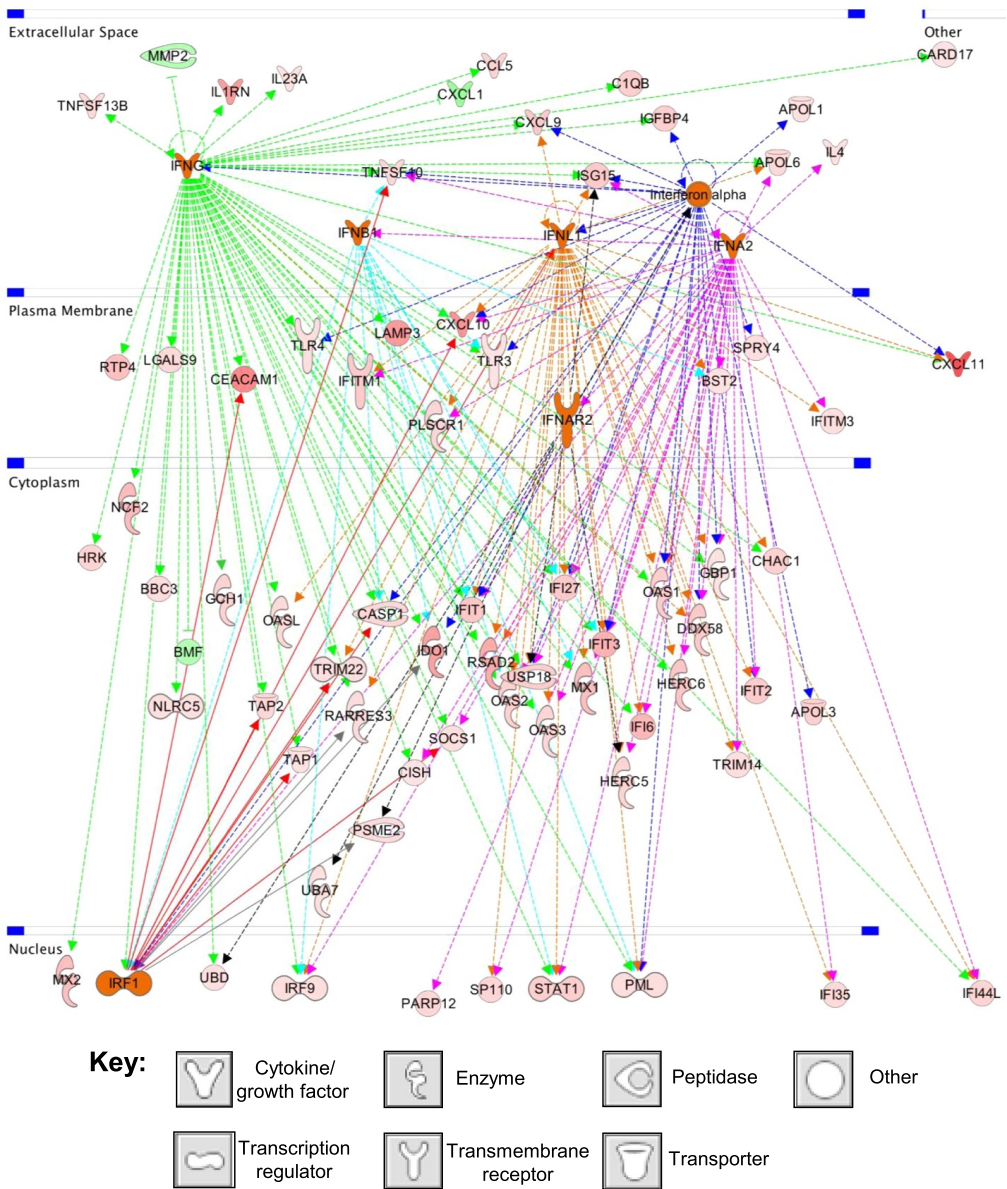
Interferon signaling upstream regulator pathway, with subcellular compartmentalization indicated. The activated upstream regulators identified (orange) include interferons IFNα (dark blue dashed lines), IFNα2 (pink dashed lines), IFNβ (teal blue dashed lines), IFNγ (green dashed lines), IFNL1 (orange dashed lines), and IRF1 (red solid lines), and regulate many immune responses. Shapes filled with deeper shades of red and green denote human tumor genes that are up regulated (red) or down-regulated (green) by metronomic CPA to a greater extent as compared to lighter shades, as indicated by microarray analysis. Key at the bottom: shapes used to indicate the molecular class of each factor.

IPA’s Upstream Regulator Analysis is a powerful way to identify putative ‘master regulators’ of complex gene expression changes, such as those induced by metronomic CPA treatment. This analysis is particularly important for upstream regulators that are regulated at the protein level (e.g., by phosphorylation, or by ligand binding) and therefore would not be identified by gene expression microarray analysis. We implemented this analysis 1) to identify upstream regulators of the U251 tumor genes showing consistent responses at both metronomic CPA time points, and 2) to determine whether the upstream regulators are *activated* or *inhibited*, based on the direction of CPA-induced responses of their gene targets. We thus identified several interferon signaling network members as the most significantly activated upstream regulators (IFNα, IFNα2, IFNβ, IFNγ, IFNL1, IRF1) (Table 3); together, these factors regulate many downstream immune response genes (Figure 2, Additional file 2: Figure S2A). Other activated upstream regulators include: TGM2 (transglutaminase 2), which is associated with glioma stem-like cells [37]; the PAF1 transcriptional complex [38]; IL27, which induces differentiation of glioma cell to astrocytes [39] and promotes anti-tumor immune responses [40] (Additional file 2: Figures S2B and S2C); growth hormone, which increases NK cell cytotoxicity to glioma cells [41]; and the endostatin precursor COL18A1. Top upstream regulators whose activity is inhibited by metronomic CPA include: MAPK1 and ERK1/2, which mediate cell proliferative signals; IL1 receptor antagonist IL1RN, which supports malignant glioma growth [42]; HIF1A (hypoxia-inducible factor-1 and EPAS1 (hypoxia-inducible factor-2 which mediate responses to hypoxia and can promote glioma growth [43]; NUPR1, which has a functional role in cancer cell resistance to conventional chemotherapeutic drugs [44]; GAPDH, which is dysregulated in several cancers, including glioma, and may promote tumor growth [45]; NEDD9, an adhesion protein that increases glioblastoma invasiveness [46]; SOCS1, a negative regulator of cytokine signaling; MAP3K7/TAK1, a key component of NFκB and MAP kinase signaling linked to the innate immune system [47]; RELA, which contribute to tumor cell survival and promotes inflammation in the tumor microenvironment [48]; and TGFB1, which increases glioma malignancy [49]. The inhibition of these upstream regulators, which primarily have pro-tumor functions, is consistent with the therapeutic effectiveness of metronomic CPA in this glioma model. Other upstream regulators did not exhibit a clear pattern of activation or inhibition (Additional file 1: Table S3).

**Table 3:** **Upstream regulators of metronomic CPA-responsive human genes**

Upstream regulator	Molecule type	p-value of overlap	# of target genes
**A. Activated upstream regulators (human gene targets)**			
IFNL1/IL29	Cytokine	1.72E-29	36
IFNG	Cytokine	5.04E-23	68
IFNA2	Cytokine	8.87E-23	36
TGM2	Enzyme	6.30E-14	43
IFNB1	Cytokine	7.98E-10	14
Interferon alpha	Cytokine	1.57E-07	23
PAF1	Transcription complex	1.89E-07	14
IRF1	Transcription regulator	1.25E-06	12
IL27	Cytokine	1.65E-06	14
Growth hormone	Protein hormone	1.37E-05	12
COL18A1	Endostatin precursor	9.08E-05	13
**B. Inhibited upstream regulators (human gene targets)**			
MAPK1	Kinase	4.88E-30	57
IL1RN	Cytokine	2.26E-15	26
HIF1A	Transcription regulator	1.09E-14	40
EPAS1	Transcription regulator	6.48E-12	24
NUPR1	Transcription regulator	3.60E-11	68
GAPDH	Enzyme	2.75E-10	14
NEDD9	Cell adhesion protein	2.34E-08	13
SOCS1	Cytokine signaling inhibitor	6.93E-08	12
MAP3K7/TAK1	Kinase	1.05E-07	12
ERK1/2	Kinase	1.47E-07	22
RELA	Transcription regulator	4.72E-07	24
TGFB1	Growth factor	6.69E-06	37

Regulators were identified by IPA of the set of U251 human tumor cell genes up-regulated or down-regulated by metronomic CPA in common at both the day 12 and day 18 times points. Shown are the upstream regulators whose activation state is reliably predicated to be activated (A) or inhibited (B) by CPA treatment, based on a bias-corrected |Z-score| >2, and that meet the stringent threshold for overlap with the target gene set at p < E-04 and contain a minimum of 10 target genes in the regulated gene set. More complete information, including Z-scores, lists of target genes for each regulator, associated mechanistic networks, and other upstream regulators are shown in Additional file 1: Table S3.

### Characterization of host (mouse) gene responses linked to immune cell activation and tumor infiltration

Next, we used mouse microarrays to investigate the extent of host (mouse) immune cell involvement in the response to metronomic CPA treatment. We analyzed mouse gene responses in U251 tumors at the same two time points analyzed on the human microarrays, and additionally, at a single time point in a rat 9L glioma model, where the innate immune response to metronomic CPA is very similar to that of U251 gliomas, but requires 1–2 additional CPA injections until robust immune cell recruitment and tumor regression become apparent [12]. Metronomic CPA treatment induced 326 mouse genes and repressed 288 mouse genes in common on all three microarrays. The consistent regulation of these 614 mouse genes in both tumor models/at all three time points indicates they are robust responses (see Additional file 1: Table S4 for full listing). Large numbers of other mouse genes were late responding genes, i.e., they did not respond to metronomic CPA until the second U251 time point (treatment day 18) and also responded significantly in 9L tumors on treatment day 24 (833 up-regulated, 823 down-regulated genes). Only 8 mouse genes showed inconsistent (i.e., opposite) patterns of regulation between the two U251 time points.

Mouse genes up-regulated in both tumor models (9L, and at least one of the U251 tumor time points) were enriched in gene clusters that include the following: immune response, lysosome, regulation of cytokine production, lectin/carbohydrate binding, cytokine receptor interaction, induction of programmed cell death, leukocyte activation, and regulation of immune effector process (Table 4A). The up-regulated gene cluster showing the second highest enrichment, immune response, includes many complement genes (*C1qa*, *C1qb*, *C1qc, C1ra, C2, Cfb, Cfd, Cfp)*, chemokines (*Ccl19, Ccl24, Ccl25, Ccl3, Ccl4, Ccl6, Ccl9, Cxcl14*), toll-like receptors (*Tlr1, Tlr4, Tlr7, Tlr8, Tlr13*), cell death effectors that act via apoptosis (Fas receptor ligand, *Fasl,* and tumor necrosis factor *Tnfsf4* and other family members and receptors *(Tnfsf10, Tnfrsf13c, Tnfrsf17)*), cytolysis (lysozymes 1 and 2, *Lyz1* and *Lyz2*), and proteolytic enzyme degradation (*Ctsa, Ctsb, Ctsd, Ctsh, Ctss*). Mouse genes that were down-regulated by metronomic CPA treatment were enriched for essential cellular functions, including DNA binding and transcription, cell division, cell migration, tube/epithelium development, and cytoskeleton organization (Table 4B). A complete listing of significant gene clusters is presented in Additional file 1: Tables S5A and S5B.

**Table 4:** **Enriched clusters of gene annotation terms for host (mouse) genes up-regulated (A) or-down-regulated (B) by metronomic CPA treatment in both U251 and 9L tumors**

Cluster name	Cluster enrichment score	Number of genes (top term)	p-value (top term)
**A. Up-regulated mouse gene clusters**			
Glycoprotein	23.1	342	5.16E-37
Immune response	13.7	87	1.11E-26
Cell surface	8.80	53	8.91E-14
Lysosome	7.38	31	2.29E-08
Extracellular membrane	6.77	191	4.07E-12
Regulation of cytokine production	6.44	28	9.78E-10
Carbohydrate binding	5.64	41	5.09E-08
Positive regulation of immune system process	4.69	41	8.70E-14
Cytokine-cytokine receptor interaction	4.10	36	1.79E-07
Lipid catabolic process	3.60	23	7.60E-07
Induction of programmed cell death	3.24	24	9.43E-06
Cell/leukocyte activation	3.22	35	6.63E-08
**B. Down-regulated mouse gene clusters**			
(Positive) regulation of transcription	10.3	71	1.06E-13
Cell division	9.31	44	4.73E-13
Repressor, negative regulation of gene expression	5.57	43	5.73E-08
Cell migration	4.81	28	6.05E-06
Spindle	4.58	20	1.22E-07
Skeletal system development	4.30	32	2.56E-06
Tube development	4.09	29	1.27E-05
Transcription factor complex	3.80	27	5.88E-06
Microtubule cytoskeleton organization	3.46	16	9.14E-05
Sequence-specific DNA binding/Homeodomain	3.04	52	4.54E-07

Analysis was based on genes that respond to metronomic CPA treatment cycles at |fold-change| >1.5 and p-value < 10^4^ at either, or both U251 treatment time points, and also in 9L tumors. Shown are clusters with enrichment scores >3.0 whose top term contains >15 genes. Also shown is the number of genes and p-value for the top term in each cluster. See Additional files 1: Table S5A and S5B for a more complete listing of significant enrichment clusters and associated gene lists.

### Host immune system-related pathways activated by metronomic CPA

**Figure 3:**
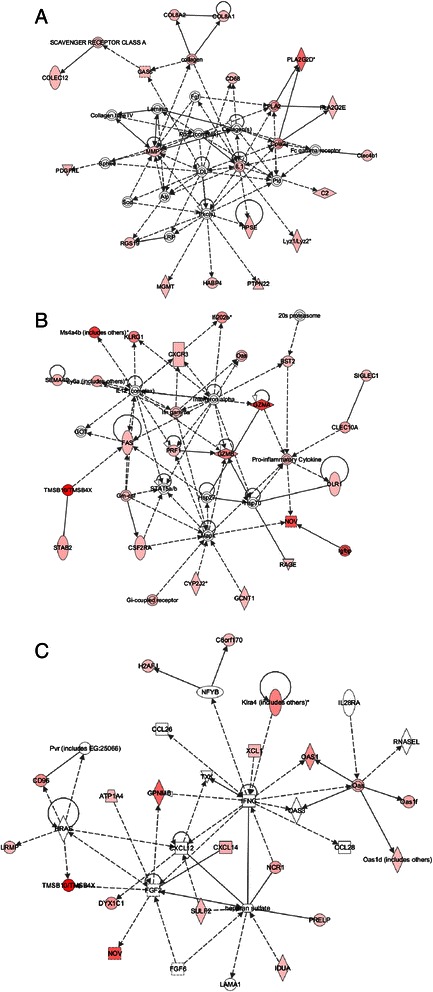
Top networks associated with mouse (host) genes induced by metronomic CPA treatment, as determined by IPA. **A)** Top network showing connections between metronomic CPA-induced expression of innate immunological disease (many macrophage-related), adhesion, infiltration, scavenger, and cytolysis genes. **B)** Top networks for NK cell-related innate immune function: tumoricidal and infectious disease NK function, targeting via CXCR3 and FAS, and cytolysis via granzyme and perforin, and **C)**, NK cell-related inflammatory disease network. Deeper shades of red indicate stronger up regulation of the gene by metronomic CPA treatment. Solid arrows: protein-DNA interactions; solid lines: protein-protein; dashed arrows: regulation of gene expression; colored: related to highlighted factor(s). Shapes indicate protein family: rectangle: receptor; square: cytokine; triangle: kinase; diamond: enzyme; oval: factor (i.e., transcription); concentric circles: complex; circle: other.

We used IPA to construct functional networks of host (mouse) genes whose expression was significantly altered by metronomic CPA treatment. Several of the networks centered on immune cell function. One network highlighted immunological disease factors, many of which are macrophage-associated, including macrophage marker *Cd68*, scavenger receptor class A molecules (i.e., *Colec12*), macrophage effector lysozymes 1 and 2 (*Lyz1* and *Lyz2*), inflammatory response phospholipases (*Pla2*, *Pla2g2d*, *Pla2g2e, Cpla2*), macrophage-produced lymphocyte mitogen *Il1*, receptor responsiveness factor *Ptpn22*, phagocytosis regulator *Gas6*, and the pro-infiltration extracellular matrix remodeling enzymes *Hpse* and *Mmp2* (Figure 3A). Two other networks grouped NK cell-related genes. The first network shows tumoricidal and infectious disease-related NK factors, including granzymes A and B (*Gzma*, *Gzmb*), perforin (*Prf1*), Fas death receptor (*Fas*), Fas-mediated apoptosis gene *Olr1*, NK cell-expressed chemokine receptor *Cxcr3* (the known interacting receptor for chemokines CXCL9, CXCL10, and CXCL11), MHC class I regulated NK marker *Klrg1*, interferon-activated genes (*Ifi202b*, *Oas1*), NK cell-expressed *Ly6a* and *Sema4d*, and lymphocyte-homing factors *Stab2* and *Nov* (Figure 3B and Figure 3C). The second network displays inflammatory disease NK cell markers NKp46 (*Ncr1*), migration factors *Cxcl14* and *Xcl1*, NK perforin cytotoxicity regulator *Klra4*, and NK adhesion molecules (*Prelp, Cd96)* (Figure 3B and Figure 3C).

### Upstream regulators of metronomic CPA-induced anti-tumor immunity

**Figure 4:**
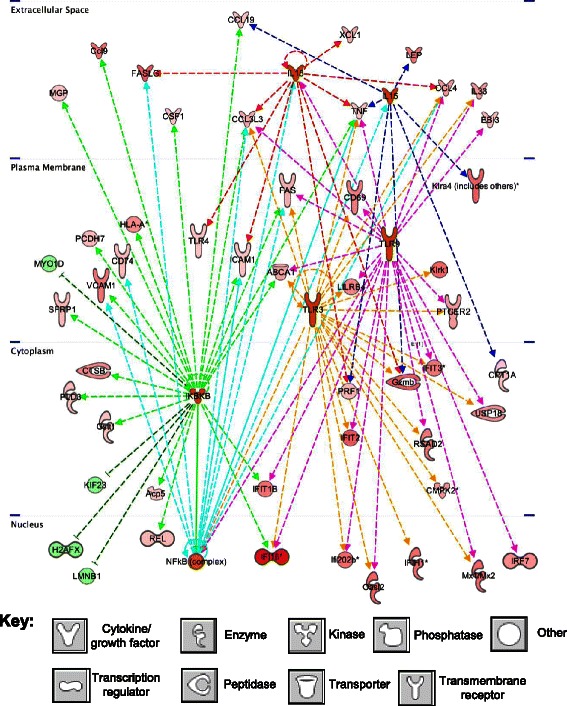
Downstream target network for the predicted upstream regulators of the mouse genes IL15 (dashed dark blue lines), IL18 (dashed red lines), TLR3 (dashed gold lines), TLR9 (dashed magenta lines), IKBKB (dashed green lines), and NFKB (dashed teal lines), showing multi-layer cell signaling and cross-talk between regulators, as well as downstream signaling and up- or down-regulation of genes identified on the mouse array as being responsive to metronomic CPA treatment. Genes and upstream regulators shown are only those that are common across tumor models at both late time points (U251 tumors on day 18 and 9L tumors on day 24). This network identifies potential signaling in tumor cell death-induced pathways, such as interferon, cytokine, tumor necrosis factor, TLR, and NFkB signaling. Deeper shades of red and green denote human tumor genes that are up-regulated (red) or down-regulated (green) by metronomic CPA to a greater extent as compared to lighter shades, as indicated by microarray analysis. Key at the bottom: shapes used to indicate the molecular class of each factor, as defined in Figure 3.

**Figure 5:**
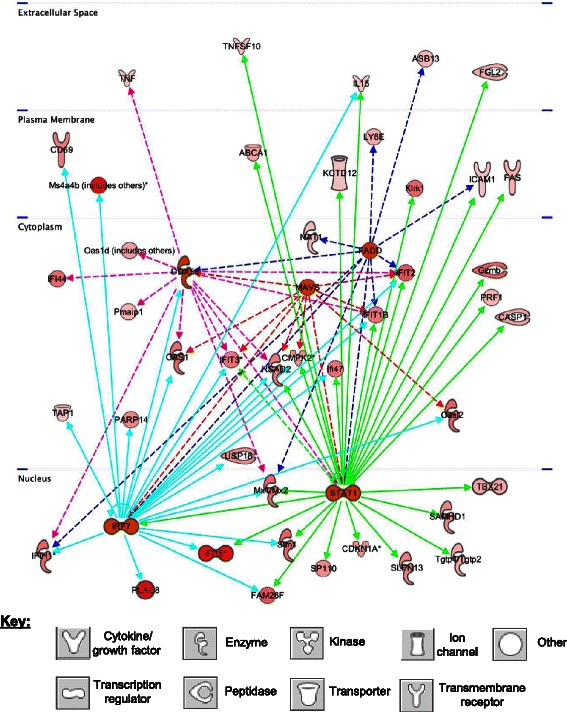
Downstream target network for the predicted mouse (host) upstream regulators DDX58 (magenta), FADD (dark blue), MAVS (red), STAT1 (green), and IRF7 (teal blue), showing multi-layer cell signaling and cross-talk amongst regulators, as well as downstream signaling and up- or down-regulation of genes identified on the mouse array following metronomic CPA treatment. Genes and upstream regulators show only those that are common to both late time points (day 18 and day 24) across the U251 and 9L models. This network identifies potential signaling in tumor cell death-induced immunogenic pathways, such as interferon, cytokine, tumor necrosis factor, and cytolysis (i.e., killer cell lectin, granzyme and perforin) signaling. Deeper shades of red and green denote human tumor genes that are up-regulated (red) or down-regulated (green) by metronomic CPA to a greater extent as compared to lighter shades, as indicated by microarray analysis. Key at the bottom: shapes used to indicate the molecular class of each factor.

We analyzed the metronomic CPA-responsive mouse (host) gene sets to elucidate potential upstream regulators, including transcription factors and cytokines that may regulate anti-tumor innate immunity induced by metronomic CPA treatment. The transcription factors PPARA, PPARG, NR1H3/LXRα and NR1H2/LXRβ were prominent as upstream regulators of mouse genes that respond to metronomic CPA early, i.e., 6 days after the 2^nd^ CPA cycle in the U251 model (Additional file 1: Table S6 and Additional file 2: Figure S3). PPARG agonists exhibit anticancer activity in glioma models [50], and increases in PPAR function have been implicated in persistent inflammation [51], while agonists of LXR promote glioblastoma cell death [52]. Upstream regulators inhibited at the same early time point include CSF2, which supports M1 macrophage polarization [53], and CD38, whose loss attenuates glioma progression [54,55] (Additional file 1: Table S6). When all mouse genes showing concordance in both tumor models at the time when CPA-induced tumor regression is well underway (i.e., treatment day 18 in U251 tumors and day 24 in 9L tumors) were considered, many more upstream regulators were identified (Table 5; Additional file 1: Table S7). The activated upstream regulators include factors related to inflammatory responses (IL6, the NF B-activating kinase IKBKB, NLRP3 inflammasome, and mir-223), interferon signaling and action (IFNAR, IFNG, IFNα/IFNβ, STAT1, IRF3, IRF5, IRF7), and TLR signaling associated with innate immune responses (TLR3, TLR4, TLR9, TICAM1, DDX58, MYD88) (Figures 4, 5, and Additional file 2: Figures S4 and S5). Other upstream regulators of the responding mouse genes include: IL12 and IL18, which stimulate macrophages and NK cells to produce IFNγ and induce glioma cell death [56]; NFATC2, which is important for inducing gene transcription during an immune response; DOCK8, required for NK cell function [57]; SASH1, whose increased expression has been related to inhibition of U251 glioma cell growth, proliferation, and invasion [58]; NOS2, a marker for M1 (anti-tumor) macrophages; BNIP3L, a pro-apoptotic factor [59]; SPI1, important for myeloid cell development an innate immunity [60]; and FADD, which is recruited to activated cell death receptors [61]. These activated upstream regulators can be considered as contributing to the antitumor actions of metronomic CPA. By contrast, another activated upstream regulator, SAMSN1, which is highly expressed in glioblastoma, is associated with poor prognosis for survival [62]. SAMSN1 has also been shown to suppress B cell activation [63]. However, we previously observed no B cell involvement in our immune competent C57BL/6, syngeneic GL261 glioma model [12]. Mouse upstream regulators inhibited by metronomic CPA in both tumor models include: CSF2 (GM-CSF), which induces differentiation of brain macrophages into M1 anti-tumor macrophages [64], but has also been shown to be neuroprotective [65], and perhaps its down-regulation is important for tumor ablation in these brain tumor models; TRIM24, which promotes glioma progression and enhances chemoresistance [66]; DNASE2; G-protein coupled receptors PTGER2 and ACKR2; SOCS1, an inhibitor of cytokine signaling; the oncomir mir-21; growth factor TGFB; and the oncogene Myc.

**Table 5:** **Upstream regulators of metronomic CPA-responsive mouse genes**

Upstream regulator	Molecule type	p-value of overlap	# of target genes
**A. Activated upstream regulators (mouse gene targets)**			
IFNAR	Interferon receptor	5.34E-14	31
IRF3	Transcription regulator	5.62E-12	28
IFNG	Interferon	2.55E-11	80
IL12 (complex)	Cytokine	1.04E-10	24
STAT1	Transcription regulator	1.32E-10	37
IRF7	Transcription regulator	1.64E-10	23
IFN alpha/beta	Interferon	3.98E-09	20
NFATC2	Transcription regulator	4.77E-09	26
IFNB1	Cytokine	9.37E-09	34
TLR4	Toll-like receptor	3.19E-08	42
DOCK8	other	4.22E-08	21
SASH1	other	7.34E-08	21
TICAM1	Adapter for TLR3	1.05E-07	28
ITK	Kinase	1.52E-07	22
SAMSN1	other	2.20E-07	23
mir-223	MicroRNA	3.67E-07	24
DDX58	Enzyme	1.15E-06	14
IL6	Interleukin 6	1.22E-06	30
SPI1	Transcription regulator	1.31E-06	19
IKBKB	Kinase that activates REL/NF B	3.41E-06	31
NOS2	Nitric oxide synthase; M1 macrophage marker	3.83E-06	30
MYD88	Adapter for TLRs	7.34E-06	32
TLR3	Transmembrane receptor	8.10E-06	27
BNIP3L	Pro-apoptotic factor	9.69E-06	15
TLR9	Transmembrane receptor	1.33E-05	25
NLRP3	Inflammasome	1.59E-05	15
IRF5	Transcription regulator	2.22E-05	12
PPARG	Ligand-dependent nuclear receptor	5.21E-05	40
IL18	Cytokine	6.86E-05	11
FADD	Transmembrane receptor adapter protein	7.33E-05	15
CD44	Enzyme	7.53E-05	22
CDKN2A	Transcription regulator	8.10E-05	20
**B. Inhibited upstream regulators (mouse gene targets)**			
CSF2/GM-CSF	Cytokine	3.82E-15	45
TRIM24	Transcription regulator	8.10E-13	32
PTGER2	G-protein coupled receptor	1.65E-09	26
DNASE2	Enzyme	4.26E-08	14
ACKR2	G-protein coupled receptor	4.87E-08	15
SOCS1	other	1.26E-07	20
mir-21	MicroRNA	1.67E-06	25
TGFB1	Growth factor	9.61E-06	19
MYC	Transcription regulator	1.46E-05	24

Upstream regulators were identified by IPA analysis of the set of mouse genes up-regulated or down-regulated by metronomic CPA in common in U251 tumors after three metronomic CPA treatments (day 18) and in 9L tumors after four metronomic CPA treatments (day 24), as described in Methods. Shown are the upstream regulators whose activation state is reliably predicated to be activated (A) or inhibited (B) by CPA treatment, with other details as described in Table 3. More complete information, including Z-scores, lists of target genes for each regulator, associated mechanistic networks, and other upstream regulators are shown in Additional file 1: Table S7.

## Discussion

Chemotherapy given as a single dose can stimulate anti-tumor immune responses, but this finding has not translated well to the cancer clinic, where many conventional chemotherapy regimens are toxic to T cells, NK cells and dendritic cells, leading to immunosuppression [67,68]. Recent studies from this laboratory have shown that administration of CPA on a metronomic, 6-day repeating schedule activates a potent anti-tumor response in several implanted glioma models leading to major regression of large tumors that is dependent on innate [12-15] as well as adaptive [12,16] immune cells. This strong therapeutic response may involve immunogenic cell death, which can be activated by CPA and several other cytotoxic anti-cancer drugs [69,70]. Here, we conducted genome-wide transcriptional profiling of implanted U251 human glioblastoma and 9L rat gliosarcoma to further characterize this response and to obtain novel insights that help elucidate the mechanisms by which metronomic CPA activates innate anti-tumor immunity.

Several of the mouse (host) gene clusters that were either activated or repressed in the tumor compartment by metronomic CPA treatment (Table 4) are similar to those found in bone marrow and spleen of tumor-bearing mice when a single CPA injection was combined with adoptive cell transfer of lymphomonocytes from tumor-vaccinated syngeneic mice [71]. Maximal anti-tumor activity against metastatic Friend leukemia 3CL-8 cells was seen when lymphomonocytes derived from spleens of the tumor-immunized mice where transferred within 24 hr after a single CPA injection, with efficacy being lost when cells were transferred after 48 hr. The authors reported a “cytokine storm” in bone marrow, and to a lesser extent in spleen and in peripheral blood mononuclear cells of the CPA-treated mice, which may contribute to the observed anti-tumor response. In the present study, we characterize the potent immune stimulatory actions of CPA in the tumor compartment of two glioma models, where intermittent administration of CPA on a 6 day repeating metronomic schedule is sufficient to activate and mobilize the host immune system, resulting in potent anti-tumor activity and major tumor regression without the need for combination with adoptive immunotherapy or immunomodulatory agents.

The present study validates our previous findings that metronomic CPA recruits and/or induces many innate immune cell factors, as seen both at the level of RNA and protein in the CPA-treated tumors [12-16]. These include the death receptor Fas, which stimulates macrophage activation [72,73] and acts as an important mediator of interactions between NK cells and cells marked for destruction [74]. Macrophage marker CD68, platelet marker PF4, dendritic cell markers CD74, implicated in DC migration [75], and CD209 (DC-SIGN), important for antigen presentation, were also increased, as were NK cell markers NKp46 (*Ncr1*) and NK1.1 (*Klrb1*), NK activating receptor NKG2D (*Klrk1*), NK effector granzymes A and B, and perforin, which are essential for cytotoxic lymphocyte-mediated cell death [76]. Cytokine and chemokine immune attractants CXCL14, IL12β and CXCL12/SDF1α were also increased by metronomic CPA treatment, implicating chemokine and cytokine gradients in stimulating leukocyte and lymphocyte migration [17,18]. These targets were all validated by host (mouse) microarray analysis and shown to be up-regulated by metronomic CPA in both U251 and 9L gliomas.

The present study also identified many additional genes and pathways that can contribute to the strong immune response activated by metronomic CPA treatment. Factors involved in immune-mediated cytolysis were identified, including the macrophage cytolytic effectors lysozymes 1 and 2 (*Lyz1* and *Lyz2*) [77], corroborated in a recent study from our group [14], as well as several other NK cell effector granzymes [74] not investigated in our earlier studies of metronomic CPA treated-gliomas. Many additional macrophage-related genes were increased in the metronomic CPA-treated tumors (Figure 3A), supporting macrophage involvement in metronomic CPA-induced tumor regression. These include *Col8A1* and *Col8A2*, collagen factors important for macrophage adhesion, and macrophage-associated phospholipases (*Pla2g2d*, *Pnpla2*), which are inducible by inflammation-induced interferon-secretion [78]. Other factors induced in the treated gliomas include scavenger receptor *Colec12,* which is important in host defense responses, matrix metalloproteinase-2 (*Mmp2*) and heparanase (*Hpse*), important for extracellular matrix remodeling and macrophage infiltration [79], *Ptpn22,* a phosphatase important for macrophage responsiveness, and *Gas6*, which regulates macrophage phagocytosis (Figure 3A). Other NK cell markers increased in the metronomic CPA-treated tumors include *Ly6a*, *Sema4d* (Cd100), *Klrg1*, an inhibitory NK cell receptor associated with memory NK cells [80], *Stab2,* which is important for lymphocyte homing and cell adhesion [81], the NK adhesion ligand *Cd96* [82], and regulators of NK cytotoxicity, such as *Sh2d1a* and *Klra5* [83,84] (Figure 3B and Figure 3C). Innate immune cell-associated genes *Tmsb10* and *Tmsb4x*, which are important for cell migration and adhesion, were strongly induced (Figure 3B, left and right). Coagulation factor 3 (*F3*), which may contribute to an immune-mediated inflammation response [85], was also significantly up-regulated in the metronomic CPA-treated tumors. Further, C3 and other complement components were significantly increased by metronomic CPA treatment in both tumor models (Additional file 2: Figure S7) and likely contribute to the observed innate immune activation and clearance of dying tumor cells [86]. Another induced complement factor, *C1q*, is synthesized by monocyte-derived macrophages and dendritic cells and is important for opsonization and forming membrane lytic complexes [87], and perhaps is employed to clear CPA-damaged tumor cells. Finally, many factors related to immune-mediated proteolysis and cell death, such as C1-esterase inhibitor (*Serping1*), lymphotoxin A (TNFβ, *Lta*), and lymphocyte cytosolic protein 2 (*Lcp2*), were identified as late responding mouse genes in the U251 and 9L models, while several cathepsins (*Cts genes*) were increased both at early and at late time points in both tumor models.

Upstream regulator analysis identified several type I and type II interferon signaling network members as being activated and most significantly associated with gene responses to metronomic CPA treatment (Tables 3 and 5). Many gene expression changes in the tumor compartment were thus linked to interferon signaling pathways (Figure 2, Additional file 2: Figure S6A and S6B) [88]. Examples include interferon--activated genes (*Oas*, *Oas1*, *Gpnmb*), genes associated with interferon-induced immune-mediated apoptosis of target cells (*Olr1*, *Pml*, *Fas*, *Fasl)* [89], and many interleukins, chemokines and cytokines involved in inflammation-based immune activation, proliferation, and mobilization, including colony stimulating factor 1 (*Csf1*), Ccl family members, Xcl1, interleukins 15 and 18 (*IL15, IL18*), and interferon-1, whose secretion can potentiate IL15 expression (Additional file 2: Figure S6B). Interferons exert many immune regulatory functions and can enhance the anti-tumor activity of CPA [90]. Upstream regulators of the tumor-associated mouse genes significantly altered by metronomic CPA treatment include DDX58, as well as FADD and MAVS. DDX58 is a pattern recognition receptor [91] that signals through FADD and MAVS (Figure 5) and may contribute to the observed anti-tumor immune responses. FADD is important for innate immune-based host defense [92] and signals through MAVS (Figure 5), which induces innate immunity by activating transcription factors IRF3 and IRF7, both of which increase type I interferon production [93]. Mouse cell-expressed TLR3 and TLR9, strongly induced by metronomic CPA, were identified by upstream regulator analysis as being involved in interferon response pathway activation (Figure 4).

Many other activated upstream regulators identified here (Tables 3 and 5) are also associated with anti-tumor immune responses. Examples include IL27, which promotes anti-tumor immunity [40], and growth hormone, which increases NK cell cytotoxicity to glioma cells [41]. Further, many of the inhibited upstream regulators identified (Tables 3 and 5) have strong pro-tumor activities (e.g., TGFB1, which increases glioma malignancy [49]), consistent with the overall strong anti-tumor responses that we have seen in the metronomic CPA-treated gliomas. However, not all of the gene expression changes induced by metronomic CPA are associated with beneficial responses. Select examples of undesirable tumor cells gene responses, shown in Table 1B, include the induction of CEACAM1, an interferon-inducible immune-inhibitory adhesion molecule [94], EREG, which induces glioma cell growth [95], and IDO1, an interferon-inducible immunosuppressive factor that is particularly active in glioma [96]. Undesirable responses involving upstream regulators of mouse genes were also observed, including activation of SAMSN1, which is associated with poor prognosis for survival in glioblastoma [62]. Further study is required to determine whether these are feedback inhibitory (compensatory) responses, and how they may impact the overall anti-tumor response to metronomic CPA treatment.

Recruitment of the innate immune system to metronomic CPA treated gliomas [12-14] is likely stimulated by tumor cell damage or stress response, with a critical level of DNA damage or cellular stress threshold being required to initiate a robust anti-tumor immune response, as suggested by the steep dose–response curve for metronomic CPA activation of an innate immune response in 9L gliomas [13]. Some of these effects are drug and tumor model dependent: 9L tumors do not begin to regress until the third cycle of every 6-day metronomic CPA treatment [11,97], U251 tumors begin to regress immediately after the first CPA injection [12], and GL261 tumors regress around the time of the second treatment cycle [12,15,16]. Since the regression of these gliomas is at least in part dependent on innate immunity, these findings suggest that anti-tumor immunity is triggered by a different drug-dependent kinetic of induced damage or stress in each tumor model. Several candidate pathways can be considered, including DNA damage, heat shock, cellular senescence, wounding and stress responses [98]. Specific examples include genes associated with oxidative stress (H2-M3, KLKR1, and TLR4; Additional file 1: Table S5A), retinoic acid (BMP4, CD38, MICB, IGFBP7, and KLF4; Additional file 1: Table S2A), and DNA damage response (p53 inducible protein *Trp53inp1*, *Mgmt*, *Casp1*, and *Casp10*; Additional file 1: Table S5A), all of which were induced by metronomic CPA treatment.

Metronomic CPA induced more than 30 genes important for induction of apoptosis, as seen in both human U251 tumor cells (human array), as well as in host cells of mice bearing U251 or 9L gliomas (mouse array) (Additional file 1: Tables S2A and S5A), consistent with our earlier finding that CPA kills 9L glioma cells by activating caspase 9-dependent apoptosis [99]. However, apoptosis-independent mechanisms of CPA-induced tumor cell death have been described, and may contribute to tumor regression [100]. Apoptosis-independent (necrotic) pathways that link macrophage-associated immunity to CPA-damaged tumors have been identified [100], and CPA-induced secretion of HMGB1 protein, a hallmark of immunogenic cell death [101] that may be a critical step in the activation of an anti-tumor immune response, independent of an intracellular apoptotic cascade [69,102], has been reported [90,100]. Of note, the NK cell-associated receptor for HMGB1, *Rage*, was induced in the metronomic CPA-treated tumors (Figure 3B and Figure 3C). Metronomic CPA treatment also induced a core set of death-related genes that may be important for innate and/or adaptive immune activation by damaged tumor cells, including *Sp110*, *Mx1*, *Mx2*, *Ebi3*, *Eomes*, *Tnfsf4*, *Gdf15*, *Ddx58*, and *Dhx58* (Figure 1A-C, Additional file 1: Tables S1 and S4 and Additional file 2: Figure S8B).

PPARγ expression was increased almost 5-fold in metronomic CPA-treated U251 tumor cells, and this could contribute to the responsiveness of these brain tumors to metronomic CPA. PPARγ is linked to many apoptosis and interferon-related genes. In particular, IPA pathway analysis associated PPARγ with *CIDEC*, a known apoptosis inducer, and with *STAT1*, which is important for interferon and killer cell immune stimulation (Additional file 2: Figure S4C), as well as interleukin-4 and cathepsin C (Additional file 2: Figure S3). PEDF, which is strongly induced in metronomic CPA-treated 9L and U251 tumors [12], induces p53-mediated apoptosis through PPAR in HUVEC cells [103]. PPAR agonists increase the anti-angiogenic activity of metronomic chemotherapy by up-regulating endothelial cell expression of CD36, a receptor that binds thrombospondin-1 and initiates the extrinsic pathway of apoptosis [104]. Furthermore, combination of low-dose trofosfamide (a CPA derivative) with the PPAR antagonist pioglitazone and a COX-2 inhibitor resulted in tumor regression or tumor growth stasis and general improvements in patient outcome [104].

Many NK cell receptor ligands are up-regulated on the surface of cancer cells in response to cellular stresses, damage or other stimuli [98], which stimulate anti-tumor immunity when detected by NK cell surface receptors, such as NKG2D and NKp46 [98,105]. In accordance with our earlier qPCR data [12], the human DNA damage response NKG2D activating ligand *MICB* was significantly increased in U251 tumors at both early (day 12) and late (day 18) time points (Figure 1C). A second human DNA damage response NKG2D ligand, *ULBP2,* was induced in the metronomic CPA-treated U251 tumors, but only at day 18, suggesting that MICB, and not ULBP2, contributes to the early onset of U251 tumor regression following metronomic CPA treatment. Mouse (host) microarray analysis showed a significant increase in the NK cell damage response receptor *CXCR3*, which binds tumor-cell specific membrane-bound activation ligands *CXCL9*, *CXCL10*, and *CXCL11* [106], all of which were also increased on the human array. Other immune-activating receptors increased by metronomic CPA treatment include pattern recognition receptors important for distinguishing self from non-self antigens, including toll-like receptors (human tumor cell-expressed TLR3 and TLR4, and mouse cell-expressed Tlrs 1, 4, 7, 8, and 13), MHC class I and class II receptors, as well as TLR-adaptor molecules, such as IRAK3 and TICAM2 (Additional file 2: Figure S8A). TLRs signal to the innate immune system to induce the death of inflamed and damaged cells [107]. Glia, neurons, and neural progenitor cells all express TLR2 and TLR4, and may contribute to the striking responsiveness of several glioma models to metronomic CPA-induced anti-tumor immunity seen in our studies. MHC class I receptors are normally expressed on all nucleated cells, however, they are most abundant on cytotoxic T and NK lymphocytes. MHC class II complexes are present on many immune cells, in particular antigen-presenting cells, such as innate immune macrophage and dendritic cells and adaptive immune B cells [108]. IPA network analysis showed a close association between interferons and MHC class I molecules, β-2-microglobulin and the antigen-processing molecule TAP (Additional file 2: Figure S8B). MHC and T-cell receptor complexes are also important for lymphocyte-mediated cytolysis via the granzyme-perforin pathway (Additional file 2: Figures S8B and S8C) [74]. While it is unclear from our array analyses which cells specifically express these receptor complexes, these complexes should be considered as possible mediators of metronomic CPA-induced anti-tumor immunity and tumor regression.

## Conclusions

The mechanisms that govern the potent therapeutic responses to the intermittent metronomic CPA used here are only partially understood, but most likely involve immunogenic cell death [69,70,109], which can stimulate dendritic cell and CD8-T cell-based immune responses leading to long-term immunity [16]. Tumors intrinsically sensitive to CPA cytotoxicity but unresponsive to metronomic CPA activation of anti-tumor immunity [27] likely undergo non-immunogenic apoptosis. While CPA and other drugs that activate immunogenic cell death are increasingly being studied in combination with immunotherapies [10,110,111], there is a pressing need for rational, mechanistic approaches to determine optimal combinations, doses and schedules. The present identification of gene signatures of metronomic CPA responses and their upstream regulators will help elucidate underlying mechanisms and facilitate the development of clinically useful biomarkers of tumor responsiveness; such markers will ultimately be required for translation to the clinic to identify clinically effective metronomic regimens and responsive patients. The gene signatures of responsiveness include many genes known to be associated with innate immune cell recruitment and activation, as well as many novel factors whose role in the actions of metronomic CPA are poorly understood. Finally, our finding that metronomic CPA also induces the expression of factors that counter the tumor regression response, including several immunosuppressive factors, raises the possibility that such factors may contribute to tumor escape from CPA-induced, immune-based regression seen in some cases [13,15], highlighting the need to identify the mechanisms involved and then develop effective mechanisms to circumvent them.

## Additional files

Additional file 1: **Comprised of one Excel workbook containing 8 Excel sheets, named Table S1 to Table S8, and provided in .xlsx format: Table S1.** Detailed presentation of human microarray data for U251 tumors on metronomic CPA treatment days 12 and 18. **Table S2**. Annotation clusters for human U251 tumor genes up-regulated (Table S2A) or down-regulated (Table S2B) on CPA treatment days 12 and 18; relates to Table 2. **Table S3**. Upstream regulators of human U251 genes; relates to Table 3. **Table S4**. Detailed presentation of mouse microarray data for metronomic CPA-treated U251 and 9L tumors. **Table S5**. Annotation clusters of mouse genes up-regulated (Table S5A) or down-regulated (Table S5B) in U251 and 9L tumors; relates to Table 4. **Table S6**. Upstream regulators of differentially expressed mouse genes in metronomic CPA-treated U251 tumors that respond to metronomic CPA early. **Table S7**. Upstream regulators of differentially expressed mouse genes in metronomic CPA-treated U251 and 9L tumors at late time points; relates to Table 5.
Additional file 2: **Comprised of one pdf file containing Figures S1 to S8: Figure S1.** Top network showing connections between metronomic CPA-induced expression of TNF and cell death-related genes, based on U251 tumor human genes increased by metronomic CPA (day 12). **Figure S2**. Upstream regulator mechanistic and downstream target pathway networks from upstream analysis, based on U251/human array, involving: A) Human IFNG and downstream STAT and NFkB signaling. B) Human IL27, an IPA-predicted upstream regulator. C) Mechanistic network for IL27 and interferon and NFkB signaling. **Figure S3**. PPARG gene network. **Figure S4**. Mouse gene upstream regulator analysis, based on U251 tumors (CPA treatment day 12). Downstream target networks for upstream regulators of responsive mouse genes: (A) IL12 complex, (B) mir-223, and (C) STAT1. **Figure S5**. Upstream regulator analysis of late-responding mouse genes, based on CPA-treatment of U251 tumors (day 18) and 9L tumors (day 24). **Figure S6**. Tumor cell activation of host innate immune system responses: A) Interferon secretion and downstream response gene pathways activated in damaged human U251 tumor cells. B) Innate immune stimulatory interleukin-15 production linked to interferon pathway activation and secretion. **Figure S7**. Canonical pathway related to host innate immune system responses to metronomic CPA-damaged tumor cells via complement component activation. **Figure S8**. Top networks involving A) tumor necrosis factor and DNA damage response genes, potentially leading to TLR or cytokine-based immune stimulation. B) *M*ajor histocompatibility complex and interferon pathway genes important for cell-to-cell signaling, immune activation, and targeted immune-mediated cell death. C) Perforin-granzyme cytolytic pathway, also related to T cell receptor (TCR) and MHC cell targeting.

## Abbreviations

BW: Body weight
CPA: Cyclophosphamide
DNA: Deoxyribonucleic acid
DC: Dendritic cell
IPA: Ingenuity pathway analysis
MAP: Mitogen-activated protein
NK: Natural killer
MDSC: Myeloid-derived suppressor cell
PPAR: Peroxisome proliferator-activated receptor
TLR: Toll-like receptor
