# Supplementary material for: Transcriptional profiling provides insights into metronomic cyclophosphamide-activated, innate immune-dependent regression of brain tumor xenografts
Source: BMC Cancer. 2015 May 8;15:375. doi: 10.1186/s12885-015-1358-y (PMC4523019; doi:10.1186/s12885-015-1358-y)

Transcriptional profiling provides insights into metronomic cyclophosphamide-activated, innate immune-dependent regression of brain tumor xenografts

Joshua C. Doloff and David J. Waxman
Department of Biology, Division of Cell and Molecular Biology, Boston University, Boston, USA

Additional file 2, comprised of Figures S1-S8

Fig. S1 – Top network showing connections between metronomic CPA-induced expression of tumor necrosis factor and cell death-related genes, associated with U251 tumor human genes increased by metronomic CPA treatment on day 12 only (early responses), as determined by IPA. Deeper shades of red indicate stronger up regulation of the gene by metronomic CPA treatment, as determined by microarray analysis.

Fig. S2 – Upstream regulator mechanistic and downstream target pathway networks from upstream analysis of the human array. A) Mechanistic network (including only upstream regulators) for human IFNG, relating its function to downstream STAT and NFkB signaling. B) Downstream target network for human IL27, an IPA-predicted upstream regulator. C) Mechanistic network for IL27 also showing its importance for interferon and NFkB signaling. Mechanistic network colors: Shapes: Orange = predicted activation, Blue = predicted inhibition; Lines: orange/blue, leads to activation/inhibition; yellow, inconsistent with state of downstream molecule; grey, not predicted. For the downstream target network in B, deeper shades of red (up regulation) and green (down regulation) indicate stronger regulation of the gene by metronomic CPA treatment.

Fig. S3 – PPARG gene network, as determined by IPA network analysis. IPA was used to elucidate important nodal connections between a suspected tumor cell-specific regulator, and ultimate cell death determination, potentially including immune activation through interleukin, cathepsin, as well as STAT and interferon-related signaling. Deeper shades of red indicate stronger up regulation of the gene by metronomic CPA treatment.

Fig. S4 – Mouse genome upstream regulator analysis (early, day 12 U251 only). Shown are downstream target networks for the following predicted upstream regulators of the up regulated and down regulated mouse genes in U251 tumors at the day 12 time point: (A) IL12 complex, (B) mir-223, and (C) STAT1.

Fig. S5 – Upstream regulator analysis of late-responding mouse genes, based on analysis of genes responding in common between U251 tumors on day 18 and 9L tumors on day 24. Downstream target networks for the following predicted upstream regulators: (A) IL12 complex, (B) mir-223, and (C) STAT1. These upstream regulators are the same as those in Fig. S4; however, one can see dynamic changes over time, as more factors become involved in each network. D) Additional downstream target nodal network for the predicted mouse (host) upstream regulator Ifnar, showing engagement of numerous immunogenic death pathways, such as interferon, tumor necrosis factor, and MHC signaling, by metronomic CPA treatment at the late time points across both models, i.e., U251 tumors

on day 18 U251 and 9L tumors on day 24. E) IPA-predicted upstream regulator interferon gamma, IFNG, target molecule network, with cellular compartmentalization, from the mouse array, in response to metronomic CPA across both models at the late time points.

Fig. S6 – Canonical pathways related to tumor cell activation of host innate immune system responses, as determined by IPA. A) Interferon secretion and downstream response gene pathways activated in damaged human U251 tumor cells. B) Innate immune stimulatory interleukin-15 production is also linked to interferon pathway activation and secretion. Deeper shades of red indicate stronger up regulation of the gene by metronomic CPA treatment.

Fig. S7 – Canonical pathway related to host innate immune system responses to metronomic CPA-damaged tumor cells via complement component activation, as determined by IPA. Many complement components were activated by metronomic CPA and are important for opsonization and targeting of dying tumor cells by the immune system. Complement complexes may also form membrane lytic complexes, also capable of eliciting tumor cell destruction and clearance. Deeper shades of red indicate stronger up regulation of the gene by metronomic CPA treatment.

Fig. S8 – Genes increased by metronomic CPA treatment involved in other potential pathways of either tumor cell-based immune activation or immune-based tumor cell clearance, as determined by IPA. A) Top network showing connections between metronomic CPA-induced expression of tumor necrosis factor and DNA damage response genes, potentially leading to TLR or cytokine-based immunostimulation. B) *Left*, Top network for major histocompatibility complex (MHC; includes genes designated HLA) and interferon pathway genes important for cell-to-cell signaling, immune activation, and targeted immune-mediated cell death. C) Canonical pathway for increased genes in the perforin-granzyme cytolytic pathway, also related to T cell receptor (TCR) and MHC cell targeting. Deeper shades of red indicate stronger up regulation of the gene by metronomic CPA treatment.

Fig. S1

Human array, gene network (cell/DNA damage)

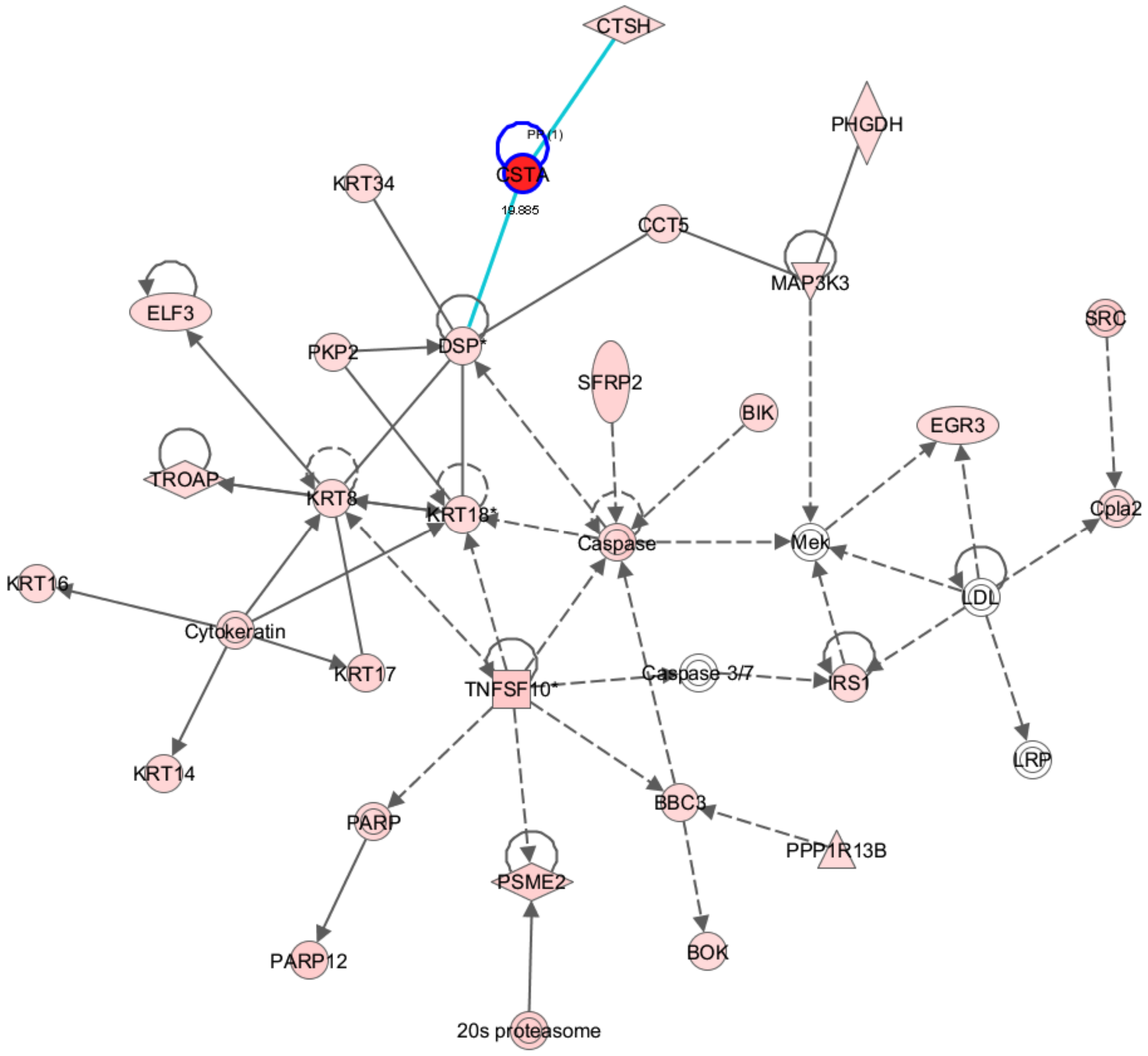

Fig. S2

Human array, upstream analysis

A

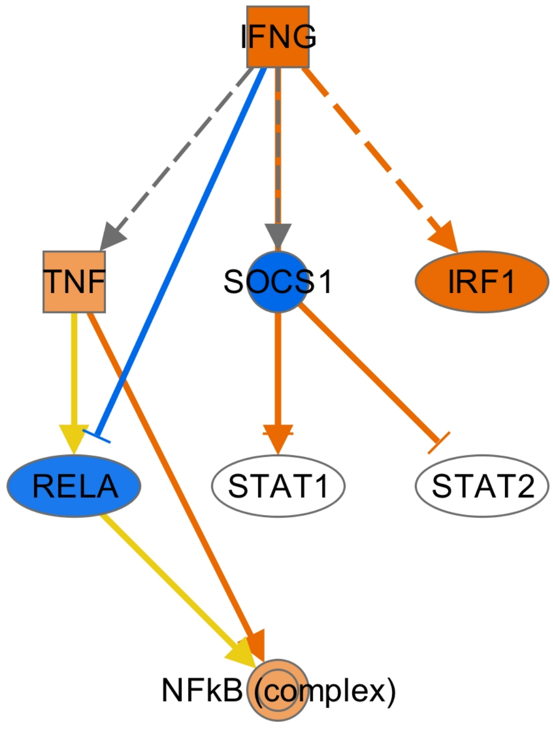

B

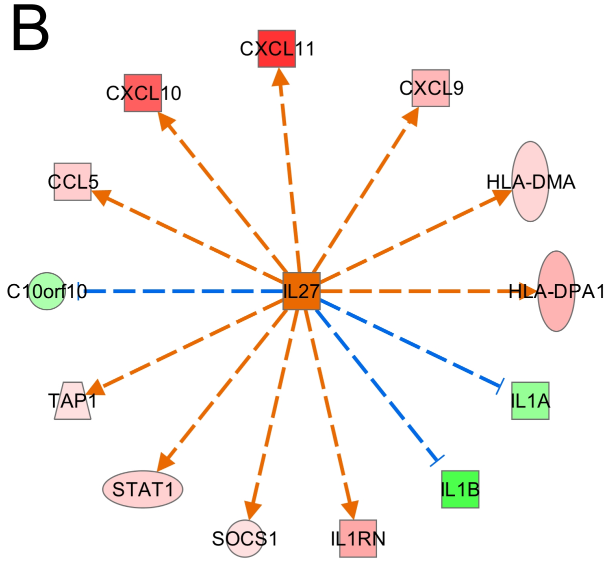

C

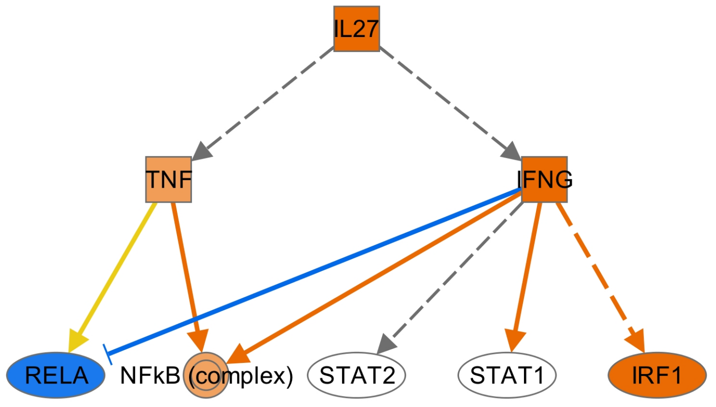

Fig. S3

Human array, gene network (PPARg-related)

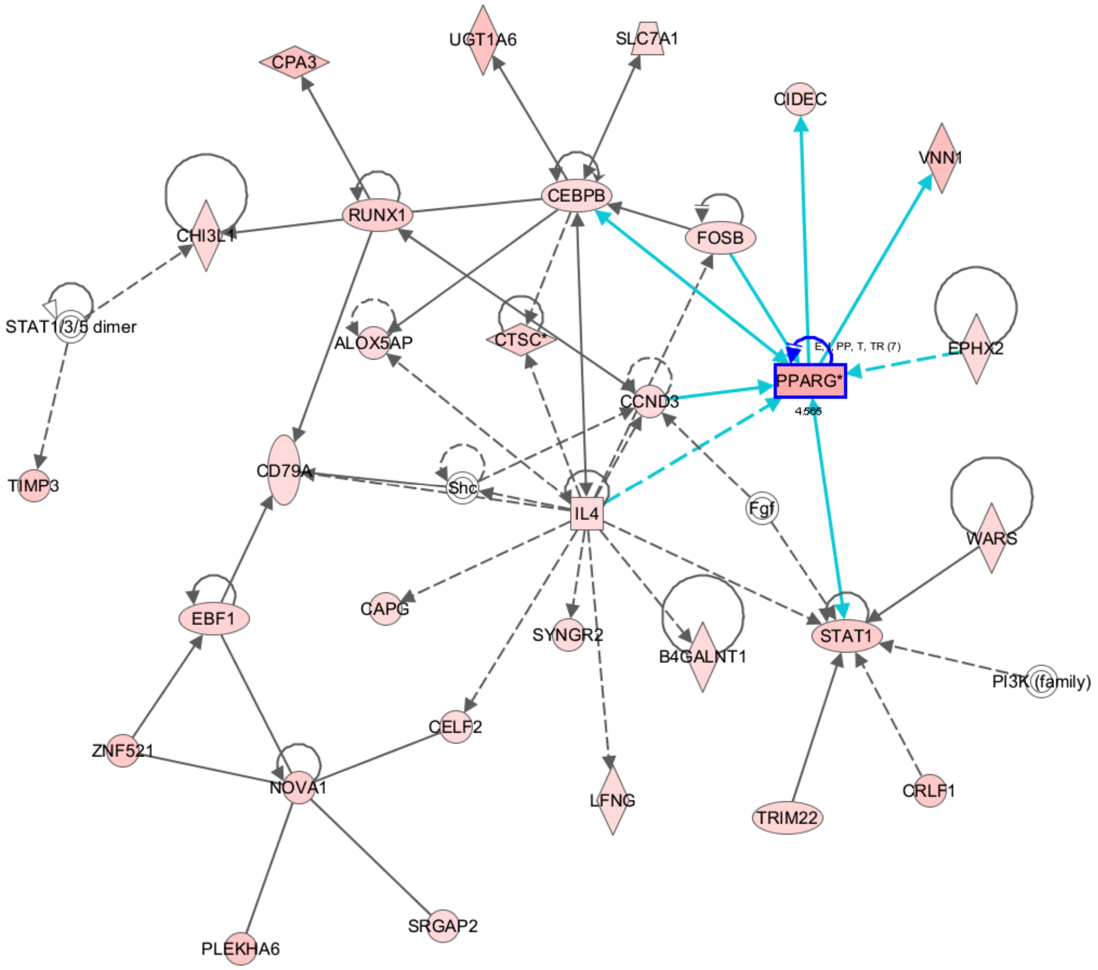

Fig. S4

Mouse up early:

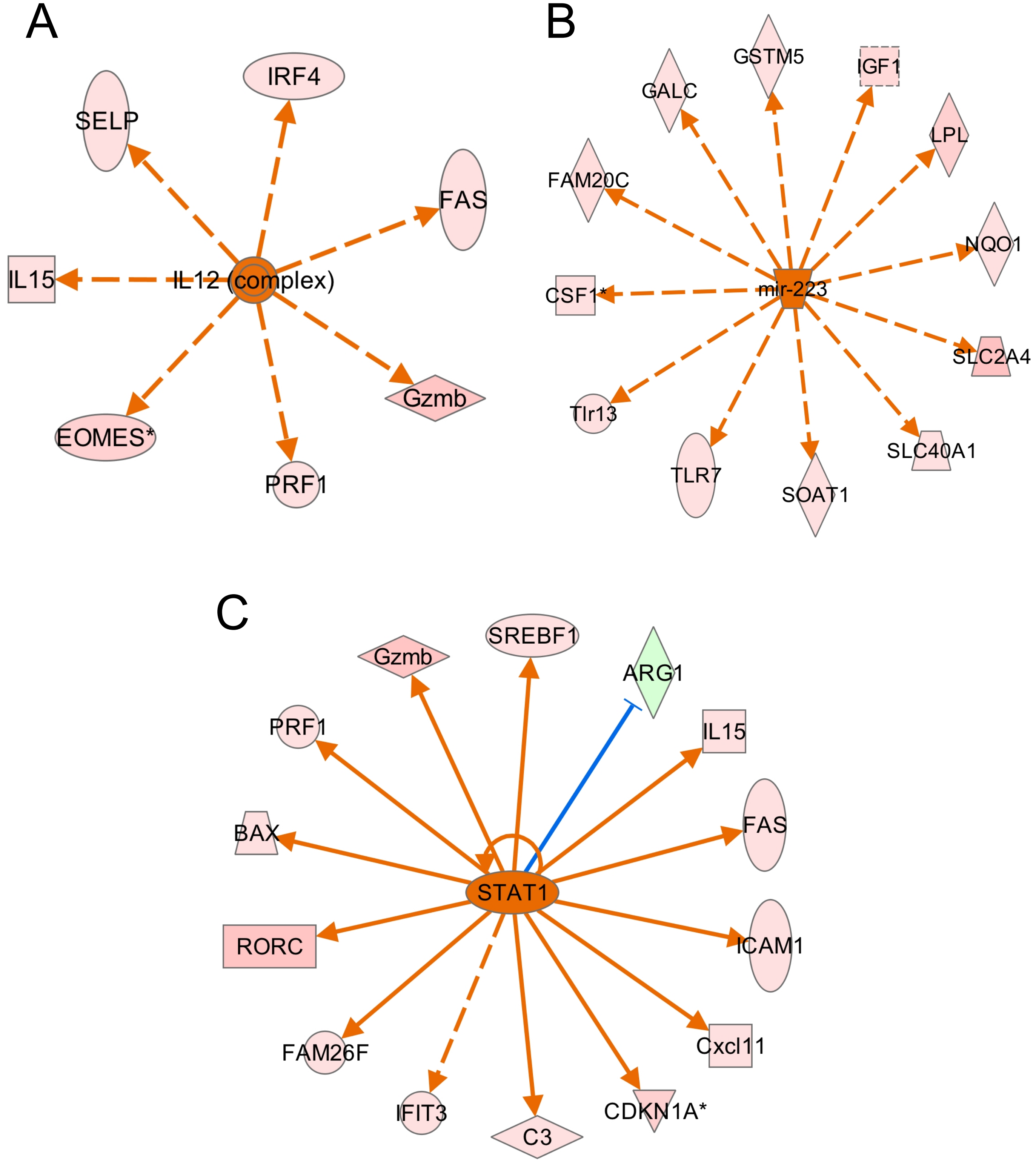

Fig. S5ABC

Mouse up late:

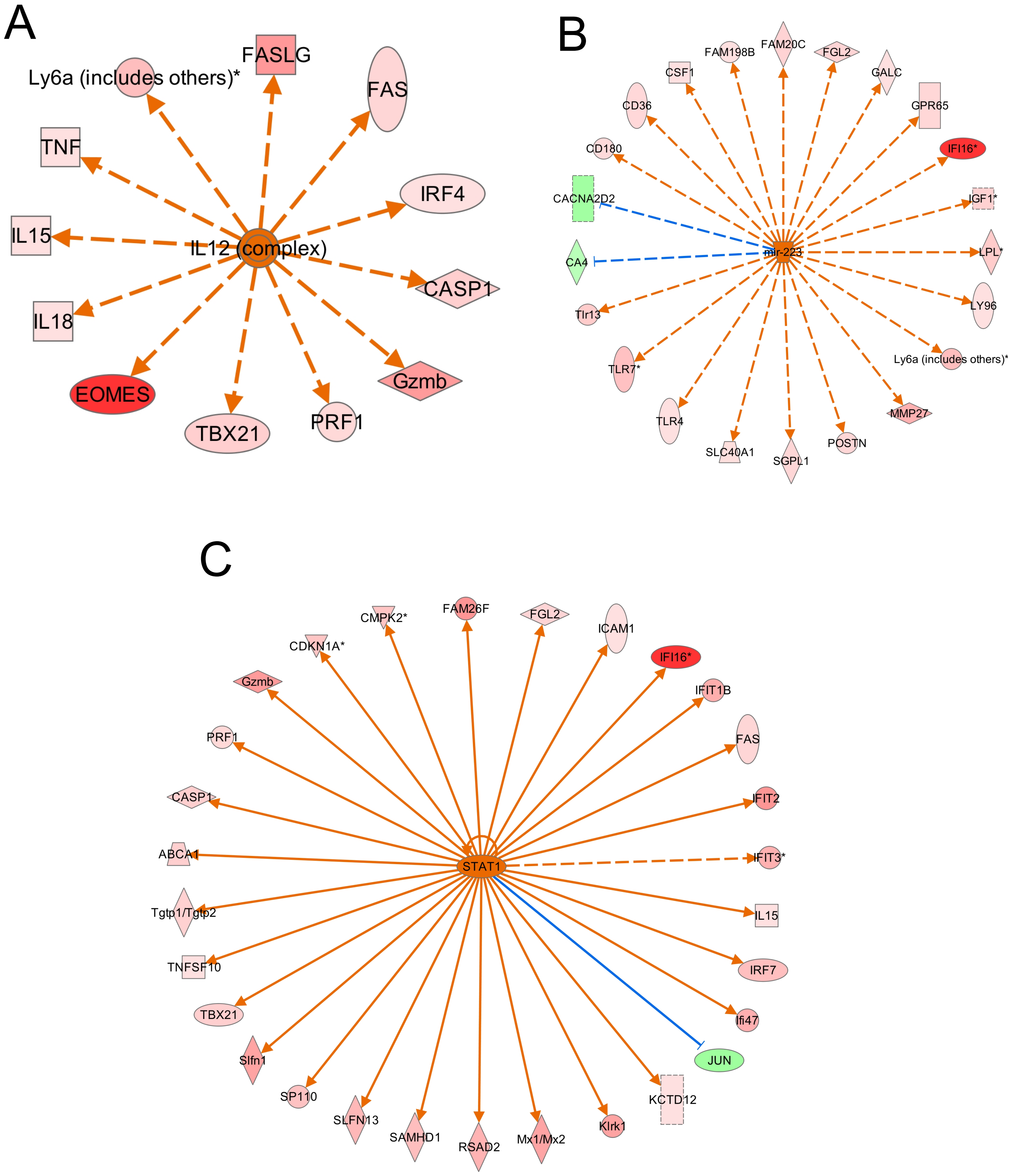

Fig. S5DE

Mouse up late (U251 & 9L):

D

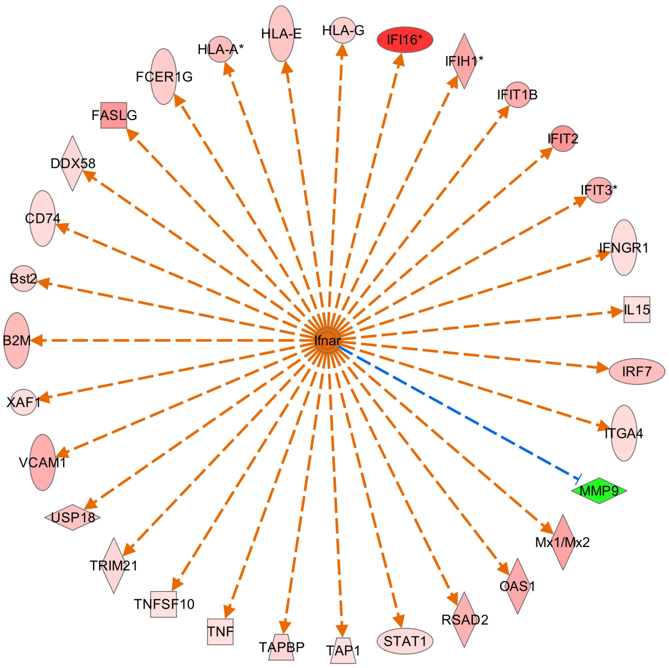

E

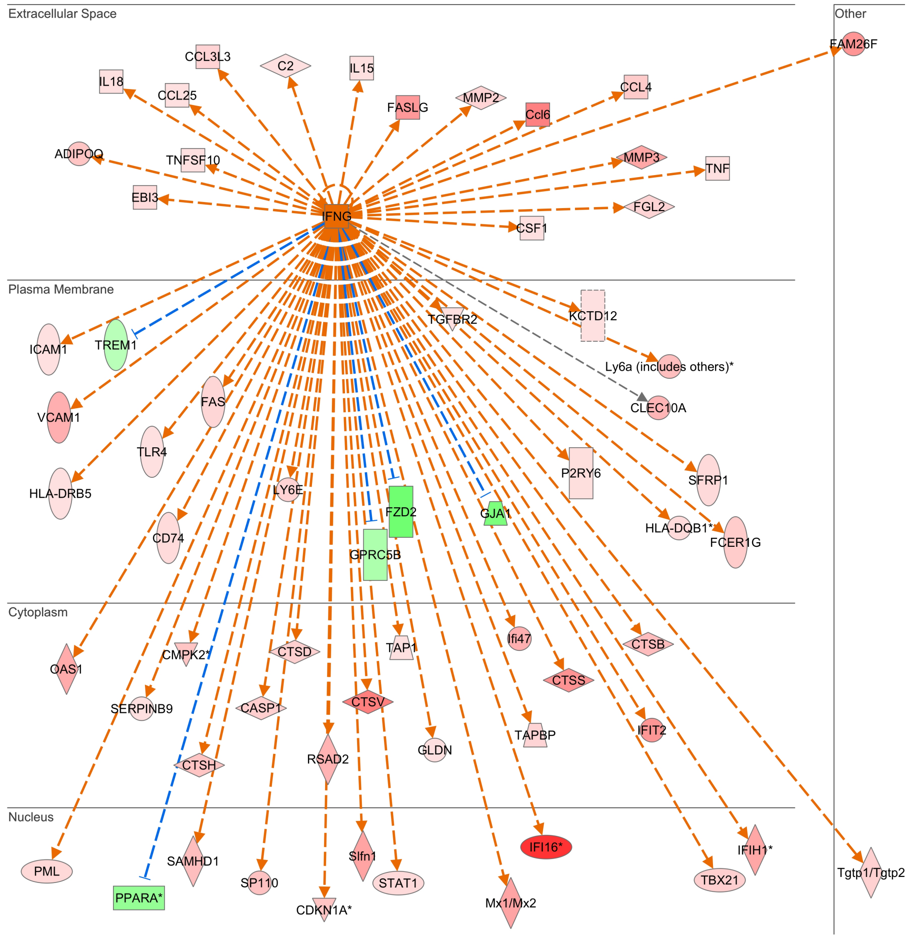

Fig. S6

A

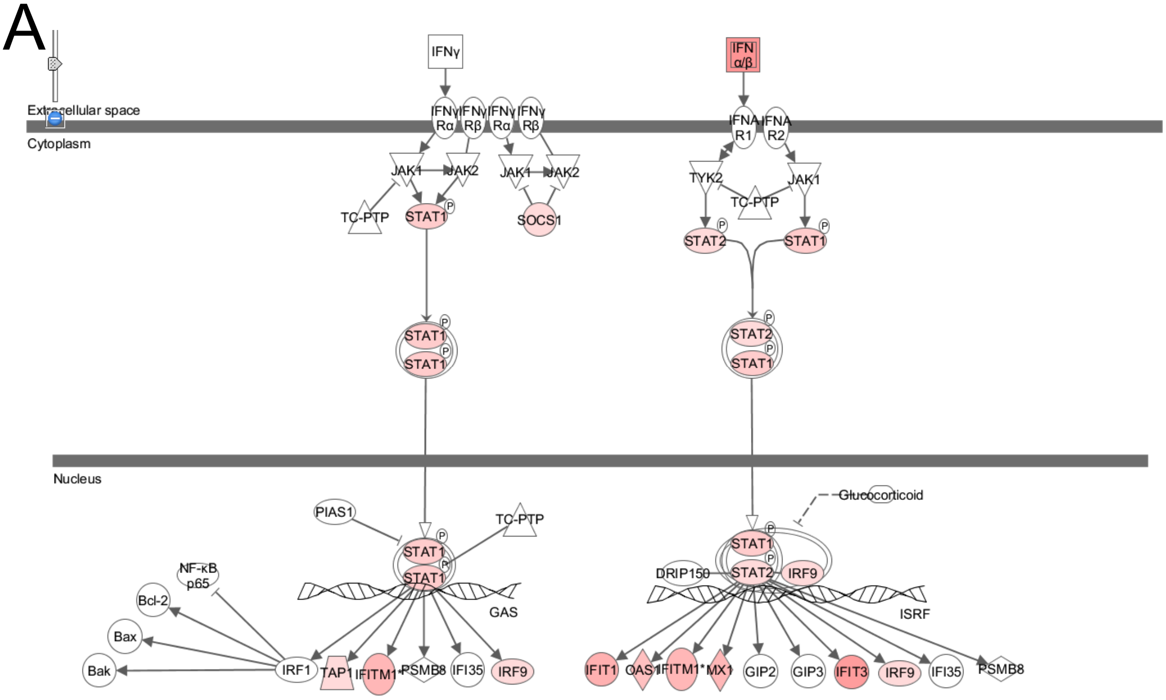
Extracellular space
Cytoplasm
IFNγ
IFNγ Rα
IFNγ Rβ
JAK1
JAK2
TC-PTP
STAT1
SOCS1
IFN α/β
IFNA R1
IFNA R2
TYK2
STAT2
Nucleus
PIAS1
NF-κB p65
Bcl-2
Bax
Bak
IRF1
TAP1
IFITM1*
PSMB8
IFI35
IRF9
GAS
Glucocorticoid
DRIP150
ISRF
IFIT1
OAS1
MX1
GIP2
GIP3
IFIT3

B

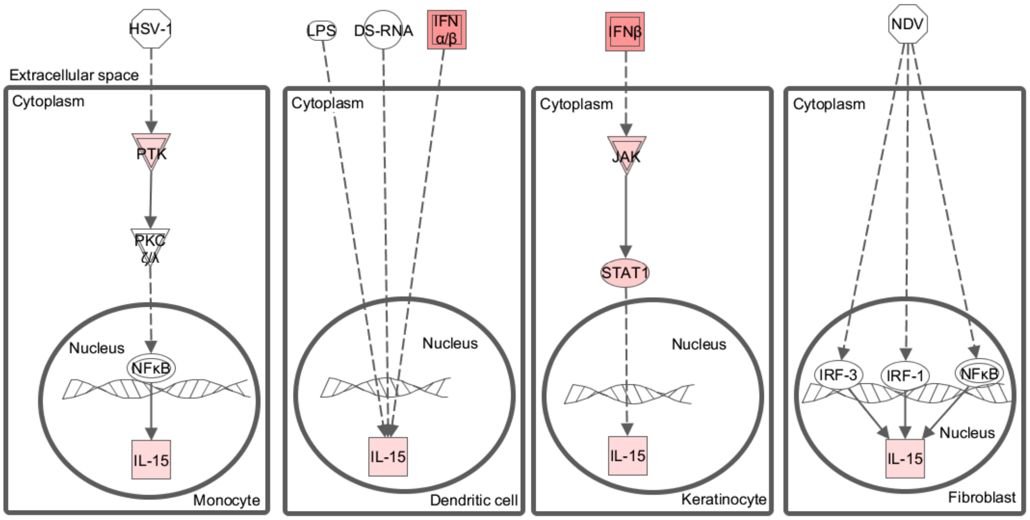
HSV-1
LPS
DS-RNA
IFN α/β
IFNβ
NDV
Extracellular space
Cytoplasm
PTK
PKC ζ/λ
Nucleus
NFκB
IL-15
Monocyte
Dendritic cell
JAK
STAT1
Keratinocyte
IRF-3
IRF-1
Fibroblast

Fig. S7

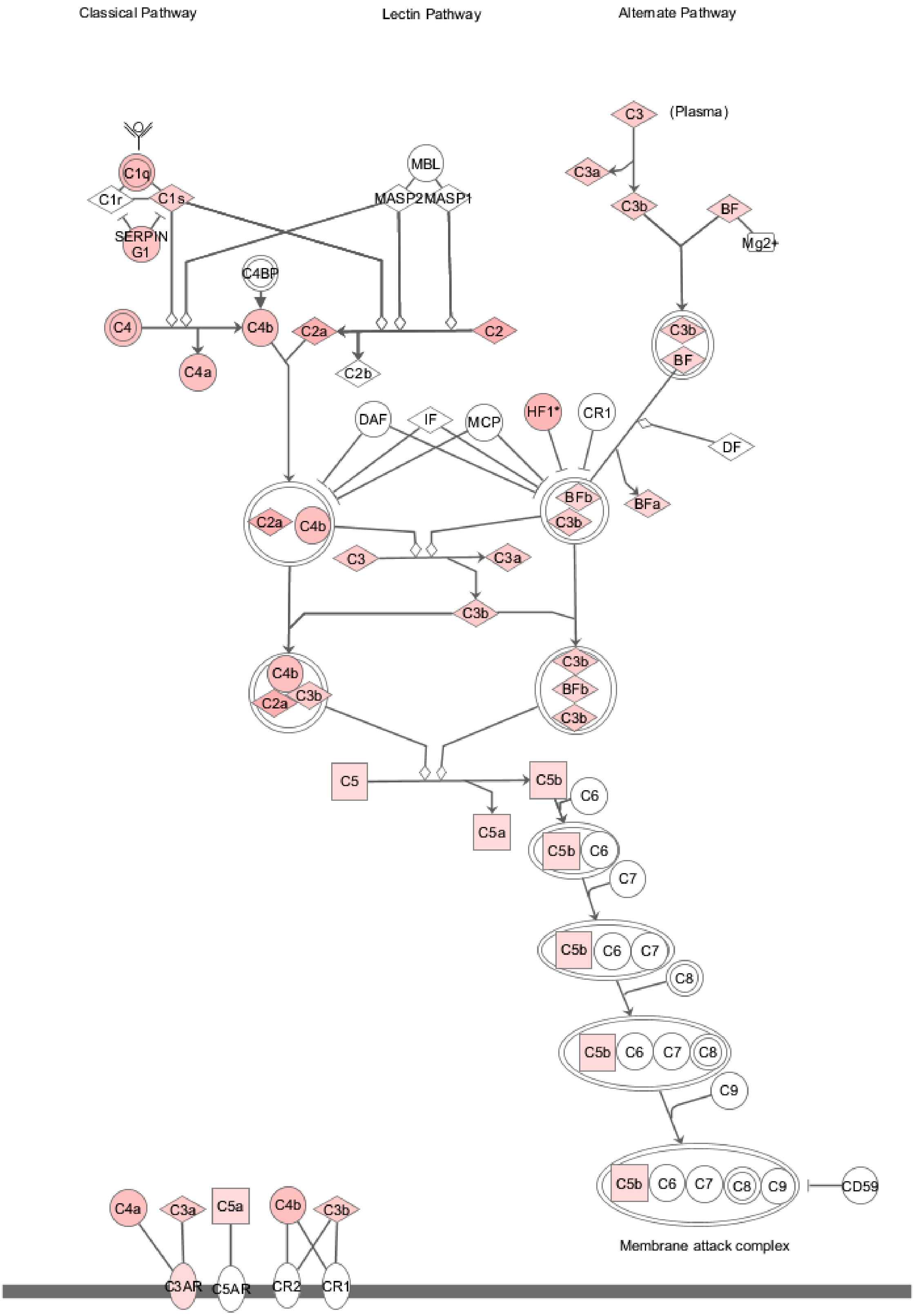
Classical Pathway
Lectin Pathway
Alternate Pathway
(Plasma)
C3
C3a
C3b
BF
Mg2+
C1q
C1r
C1s
SERPIN G1
MBL
MASP2
MASP1
C4BP
C4
C4b
C2a
C2
C4a
C2b
DAF
IF
MCP
HF1*
CR1
DF
BFb
BFa
C5
C5b
C5a
C6
C7
C8
C9
CD59
Membrane attack complex
C3AR
C5AR
CR2

Fig. S8

A TNFα/Dmg response-related

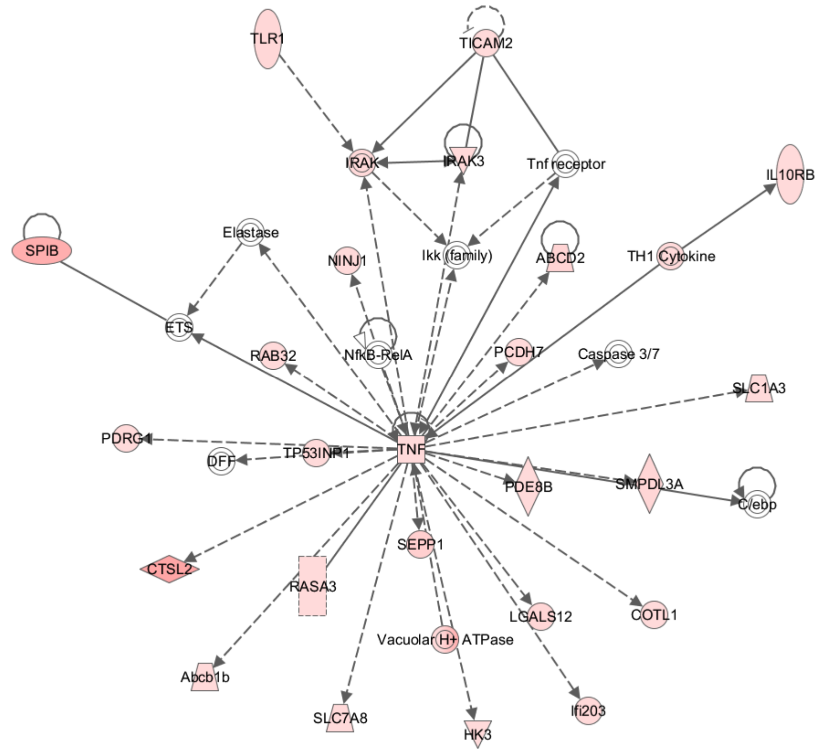

B Interferon & MHC-related

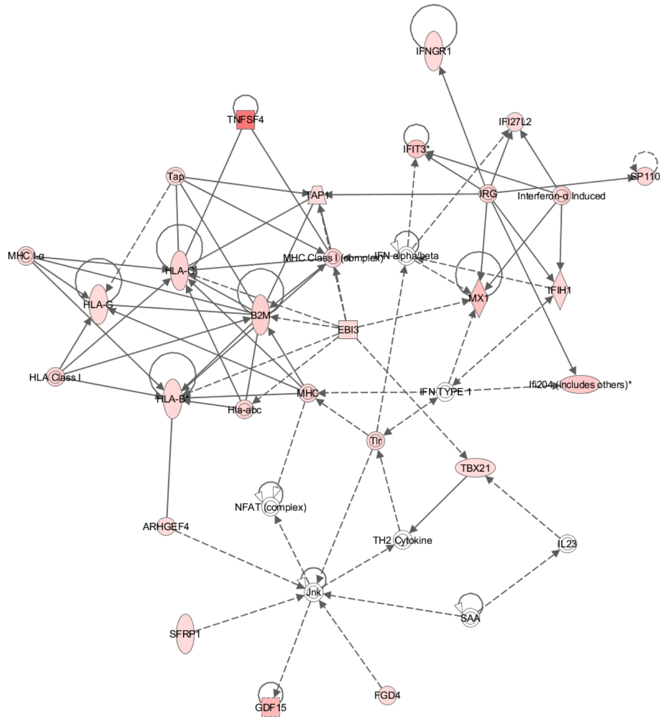

C Targeted cytolysis

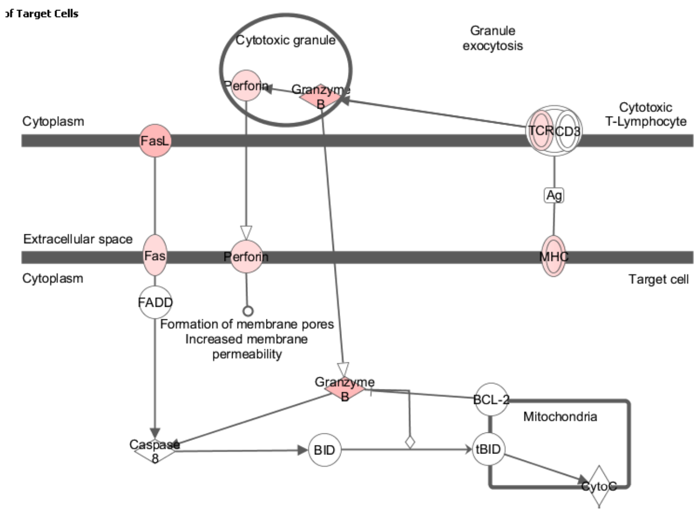

Supplement: Additional file 2: — Comprised of one pdf file containing Figures S1 to S8: Figure S1. Top network showing connections between metronomic CPA-induced expression of TNF and cell death-related genes, based on U251 tumor human genes increased by metronomic CPA (day 12). Figure S2. Upstream regulator mechanistic and downstream target pathway networks from upstream analysis, based on U251/human array, involving: A) Human IFNG and downstream STAT and NFkB signaling. B) Human IL27, an IPA-predicted upstream regulator. C) Mechanistic network for IL27 and interferon and NFkB signaling. Figure S3. PPARG gene network. Figure S4. Mouse gene upstream regulator analysis, based on U251 tumors (CPA treatment day 12). Downstream target networks for upstream regulators of responsive mouse genes: (A) IL12 complex, (B) mir-223, and (C) STAT1. Figure S5. Upstream regulator analysis of late-responding mouse genes, based on CPA-treatment of U251 tumors (day 18) and 9L tumors (day 24). Figure S6. Tumor cell activation of host innate immune system responses: A) Interferon secretion and downstream response gene pathways activated in damaged human U251 tumor cells. B) Innate immune stimulatory interleukin-15 production linked to interferon pathway activation and secretion. Figure S7. Canonical pathway related to host innate immune system responses to metronomic CPA-damaged tumor cells via complement component activation. Figure S8. Top networks involving A) tumor necrosis factor and DNA damage response genes, potentially leading to TLR or cytokine-based immune stimulation. B) Major histocompatibility complex and interferon pathway genes important for cell-to-cell signaling, immune activation, and targeted immune-mediated cell death. C) Perforin-granzyme cytolytic pathway, also related to T cell receptor (TCR) and MHC cell targeting. [file 12885_2015_1358_MOESM2_ESM.pdf]
